# Bioactive Benzofuran Derivatives from Cortex Mori Radicis, and Their Neuroprotective and Analgesic Activities Mediated by mGluR_1_

**DOI:** 10.3390/molecules22020236

**Published:** 2017-02-08

**Authors:** Ya-Nan Wang, Mao-Feng Liu, Wei-Zhen Hou, Rui-Ming Xu, Jie Gao, An-Qi Lu, Mei-Ping Xie, Lan Li, Jian-Jun Zhang, Ying Peng, Li-Li Ma, Xiao-Liang Wang, Jian-Gong Shi, Su-Juan Wang

**Affiliations:** 1 State Key Laboratory of Bioactive Substance and Function of Natural Medicines, Institute of Materia Medica, Chinese Academy of Medical Sciences & Peking Union Medical College, Beijing 100050, China; wangyanan@imm.ac.cn (Y.-N.W.); 13241468601@163.com (M.-F.L.); houweizhen@imm.ac.cn (W.-Z.H.); rmxu@imm.ac.cn (R.-M.X.); angellu@imm.ac.cn (A.-Q.L.); xiemeiping2016@sina.com (M.-P.X.); lilan@imm.ac.cn (L.L.); jjzhang@imm.ac.cn (J.-J.Z.); ypeng@imm.ac.cn (Y.P.); wangxl@imm.ac.cn (X.-L.W.); shijg@imm.ac.cn (J.-G.S.); 2 GRU Cancer Center, Augusta University, Augusta, GA 30912, USA; jgao@augusta.edu; 3 Editorial Department, Shenyang Pharmaceutical University, Shenyang 110016, China; malili022406@sina.com

**Keywords:** benzofuran-type stilbenes, neuroprotection, analgesia, Cortex Mori Radicis

## Abstract

Four new benzofuran-type stilbene glycosides and 14 known compounds including 8 benzofuran-type stilbenes and 6 flavonoids were isolated from the traditional Chinese medicine, Cortex Mori Radicis. The new compounds were identified as (9*R*)-moracin P 3′-*O*-α-l-arabinopyranoside (**1**), (9*R*)-moracin P 9-*O*-β-d-glucopyranoside (**2**), (9*R*)-moracin P 3′-*O*-β-d-glucopyranoside (**3**), and (9*R*)-moracin O 10-*O*-β-d-glucopyranoside (**4**) based on the spectroscopic interpretation and chemical analysis. Three benzofuran-type stilbenes, moracin O (**5**), R (**7**), and P (**8**) showed significant neuroprotective activity against glutamate-induced cell death in SK-N-SH cells. In addition, moracin O (**5**) and P (**8**) also demonstrated a remarkable inhibition of the acetic acid-induced pain. The molecular docking with metabotropic glutamate receptor 1 (mGluR_1_) results indicated that these neuroprotective benzofuran-type stilbenes might be the active analgesic components of the genus *Morus*, and acted by mediating the mGluR_1_ pathway.

## 1. Introduction

Cortex Mori Radicis, the root bark of some *Morus* species (e.g., *M. alba*, *M. mongolica*, *M. cathayana*, and *M. australis*) have been used in traditional Chinese medicine as an antidiabetic, diuretic, and expectorant agent. Many compounds were identified from *Morus* plants, such as Diels-Alder-type adducts, stilbenes, flavonoids, and alkaloids. Their antioxidant [1,2,3], anti-inflammatory [4,5], antimicrobial [6,7,8,9], anticarcinogenic [10,11,12], and antidiabetic [13] activities were reported frequently. Recently, the structures and bioactivities of polyphenols from *Morus* plants [14] and bioactive benzofurans [15] were reviewed. It should be noted that the extracts of some twigs [16], leaves [17], or root barks [18] of *Morus* species were used to relieve pain in folk medicine. To the best of our knowledge, only two compounds from genus *Morus*, *cis*-mulberroside A [19] and morusin [20], have been reported with analgesic activity to date, which is related to the traditional antirheumatic usage of Cortex Mori Radicis.

l-Glutamate is the major excitatory neurotransmitter in the central nervous system (CNS) and glutamate-mediated excitotoxicity plays a crucial role in neurodegenerative disorder, particularly in Parkinson’s disease, Alzheimer’s disease, epilepsy, spinal cord trauma, and ischemic stroke. As glutamate receptors are also found in peripheral tissue, they may be implicated in persistent or chronic pain, including inflammatory or joint-related pain (e.g., rheumatoid arthritis, osteoarthritis) and neuropathic pain resulting from injury and/or diseases of central (e.g., spinal cord injury) or peripheral nerves (e.g., diabetic neuropathy, radiculopathy) [21,22].

Neuroprotective effect of stilbenes, including piceid and resveratrol, has been widely reported [23], but the applications of benzofuran stilbene on CNS diseases [24] and analgesia were rarely studied. In this paper, we report four new benzofuran stilbene glycosides (**1*–*4**) and the neuroprotective and analgesic activities of their aglycones. Three benzofuran stilbenes showed significant protective activities against glutamate-induced neurotoxicity. Correspondingly, they also exhibited remarkable analgesic activities by inhibition of the acetic acid-induced pain. Structural determinations of compounds were carried out on the basis of spectroscopic data analyses. The primary action mechanism of neuroprotection and analgesia was also discussed with the help of the molecular docking with metabotropic glutamate receptor 1 (mGluR_1_).

## 2. Results and Discussion

### 2.1. Structural Analysis

**Figure 1:**
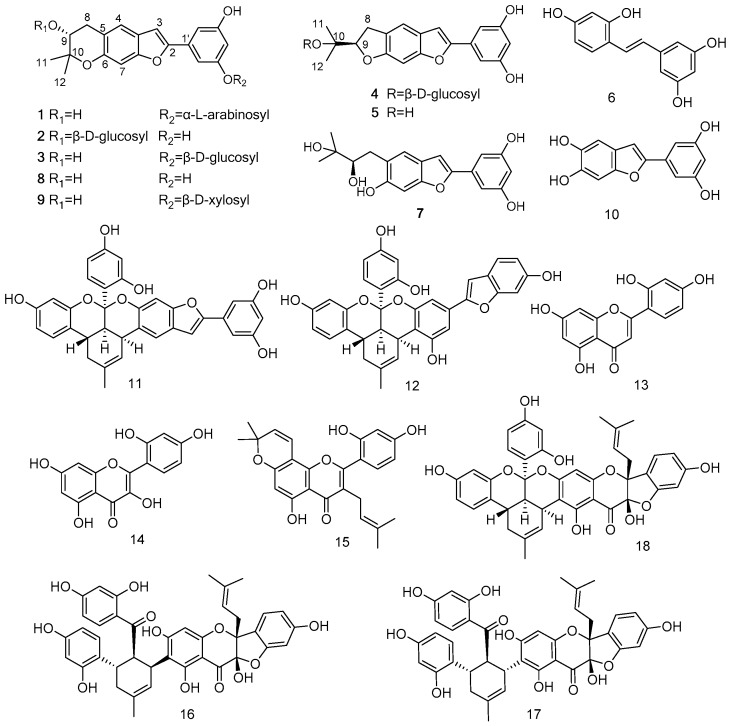
Structures of isolated compounds from Cortex Mori Radicis.

Eighteen compounds, including four new (**1**–**4**) and eight known (**5**–**12**) stilbenes and six known flavonoids (**13**–**18**), were isolated from the EtOAc-soluble fraction of Cortex Mori Radicis (Figure 1). The known compounds were elucidated as moracin O (**5**) [25], oxyresveratrol (**6**) [26], moracin R (**7**) [27], moracin P (**8**) [25], mulberroside C (**9**) [28], wittifuran E (**10**) [28], isomulberrofuran G (**11**) [29], mulberrofuran G (**12**) [30], norartocarpetin (**13**) [31], morin (**14**) [32], morusin (**15**) [33], sanggenon C (**16**) [34], sanggenon D (**17**) [35], and cathayanin B (**18**) [36] by comparison with spectroscopic data in the literatures.

Compound **1**, purified as a brown powder, had the molecular formula of C_24_H_26_O_9_ as established by HRESIMS *m/z* 459.1663 [M + H]^+^ (calcd for C_24_H_27_O_9_^+^, 459.1650). The ^1^H-NMR spectrum (Table 1) demonstrated a 2-arylbenzofuran skeleton with three *meta*-aromatic protons at δ_H_ 6.91 (1H, d, *J* = 2.1 Hz, H-2′), 6.89 (1H, d, *J* = 2.1 Hz, H-6′), and 6.43 (1H, t, *J* = 2.1 Hz, H-4′); two *para*-aromatic protons at δ_H_ 7.29 (1H, s, H-4), 6.93 (1H, s, H-7), and one singlet at δ_H_ 7.17 (1H, s, H-3). Two *gem*-dimethyl signals at δ_H_ 1.31 (3H, s, H-11), and 1.18 (3H, s, H-12), one methylene at δ_H_ 3.02 (1H, dd, *J* = 16.4, 6.0 Hz, H-8a), and 2.72 (1H, dd, *J* = 16.4, 7.0 Hz, H-8b) indicated the presence of an isoprenyl fragment. The remaining signals at δ_H_ 4.85 (1H, d, *J* = 6.7 Hz, H-1″) and δ_H_ 2.60–3.80 were assigned to a pentose moiety. Besides the five pentosyl signals, the ^13^C-NMR spectrum (Table 2) confirmed the presence of isoprenylated 2-arylbenzofuran skeleton including 14 olefinic carbons and 5 alkyl carbons. Further, HMBC correlations between H_2_-8 (δ_H_ 3.02, 2.72) and C-4 (δ_C_ 120.9) and C-6 (δ_C_ 151.2); H-9 (δ_H_ 3.67) and C-5 (δ_C_ 117.2) were observed, and these data elucidated the aglycone of **1** as moracin P [25]. The linkage of pentose was determined to be at the C-3′ position, deduced from the HMBC correlation between H-1″ (δ_H_ 4.85) and C-3′ (δ_C_ 158.8). In ^1^H-NMR spectra, the H-4″ of the pentose was a broad singlet and the coupling constants between H-4″ and H-5″, H-4″ and H-3″ were less than 4 Hz, indicated that H-4″ was equatorial. Subsequent acid hydrolysis identified the pentose as l-arabinose and the aglycone as (9*R*)-moracin P. The C-4″ (δ_C_ 68.0) indicated the arabinose was a pyranose [37]. Therefore, the chemical structure of **1** was established as (9*R*)-moracin P 3′-*O*-α-l-arabinopyranoside.

The molecular formulas of compounds **2** and **3** were both C_25_H_28_O_10_, established by HRESIMS (*m/z* 511.1588 [M + Na]^+^, calculated for C_25_H_28_O_10_Na^+^, 511.1575). Comparing ^1^H-NMR and ^13^C-NMR spectra (Table 1 and Table 2) of compounds **2** and **3** with those of compound **1**, the structures of **2** and **3** were both identified as moracin P glycosides with a hexose in their structures, and further supported by HMBC analysis. The linkage of sugar in **2** was determined to be at C-9 position, deduced from the HMBC correlation between H-1″ (δ_H_ 4.33, d, *J* = 7.7 Hz) and C-9 (δ_C_ 73.4). The sugar in **3** was connected to C-3′ position, deduced from the HMBC correlation between H-1″ (δ_H_ 4.83 d, *J* = 7.6 Hz) and C-3′ (δ_C_ 159.1). Subsequent acid hydrolysis identified the aglycone as (9*R*)-moracin P and the sugar as d-glucose. Therefore, the structures of **2** and **3** were established as (9*R*)-moracin P 9-*O*-β-d-glucopyranoside (**2**) and (9*R*)-moracin P 3′-*O*-β-d-glucopyranoside (**3**), respectively.

The molecular formula of compound **4** was established to be C_25_H_28_O_10_ by HRESIMS, which showed the quasimolecular ion peak at *m/z* 511.1589 [M + Na]^+^ (calcd for C_25_H_28_O_10_Na^+^, 511.1575). The ^1^H-NMR and ^13^C-NMR spectroscopic data (Table 1 and Table 2) of **4** suggested that it was a stilbene glucoside similar to **2** and **3**, except the dihydropyran ring. HMBC correlations between H_2_-8 (3.16, 3.35) and C-4 (δ_C_ 116.1) and C-6 (δ_C_ 158.1); H-9 (δ_H_ 4.75) and C-5 (δ_C_ 124.3) and C-6 (δ_C_ 158.1) were observed, and the aglycone of **4** was elucidated as moracin O [25]. The linkage of sugar was determined to be at the C-10 position, deduced from the HMBC correlation between H-1″ (δ_H_ 4.42, d, *J* = 7.8 Hz) and C-10 (δ_C_ 77.1). Acid hydrolysis identified the aglycone as (9*R*)-moracin O and the sugar as d-glucose. Therefore, the structure of **4** was established as (9*R*)-moracin O 10-*O*-β-d-glucopyranoside.

There was only one chiral carbon C-9 in aglycones of compounds **1**–**4**. In order to determine their absolute configuration, compounds **1**–**4** were hydrolyzed by HCl, and the aglycones were partitioned by EtOAc. After drying the EtOAc-soluble fraction in vacuo, the residue was dissolved in MeOH to determine the optical rotation. All aglycones showed negative optical rotation values (see Supplementary Materials), which confirmed that aglycones **1a**–**3a** were (9*R*)-moracin P [38] and aglycone **4a** was (9*R*)-moracin O [38,39].

**Figure 2:**
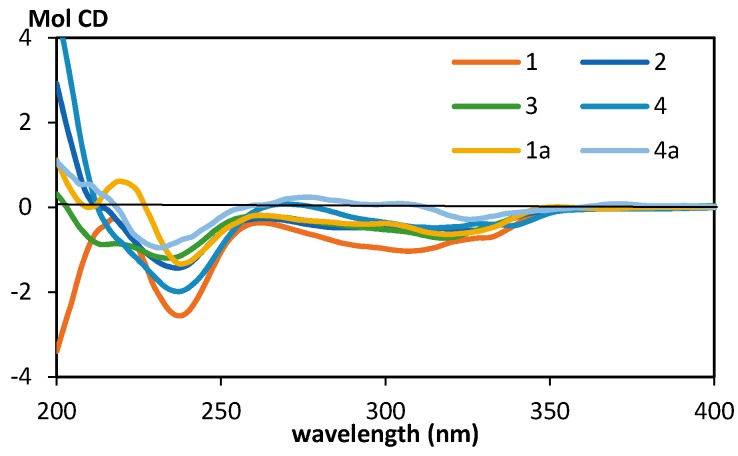
Electronic circular dichroism (ECD) spectra of **1–4**, **1a,** and **4a**.

Although the sugars were substituted at different position, the ECD (electronic circular dichroism) spectra of **1**–**4** and their aglycones **1a** and **4a** were quite similar (Figure 2). There were two Cotton effects in their ECD spectra. The broad negative Cotton effect over 280–340 nm was regarded as π→π* transition from MO86 to MO87 of 2-benzylbenzofuran. The negative Cotton effect at 240 nm was generated by π→π* transition from MO86 to MO90 of benzofuran moiety and π→π* transition from MO86 to MO89 of benzyl moiety, according to the MO analysis in the calculated ECD of **1a** (Supplementary Materials). It seemed that the chiral carbon on the rigid skeleton attached to the benzofuran affected the Cotton effect at 240 nm more than the chiral center on the flexible benzyl moiety, deduced from the similar ECD spectra of **1**–**4**, **1a**, and **4a**.

### 2.2. Neuroprotective Activities

Twelve compounds were evaluated by the 3-(4,5-dimethylthiazol-2-yl)-2,5-diphenyltetrazolium bromide (MTT) assay for the neuroprotective effects against glutamate-induced cellular damage on the human neuroblastoma SK-N-SH cells (Table 3). Moracin O (**5**), R (**7**), and P (**8**) exhibited significant protection with 40%–60% of cell viability at the concentration of 10 μM. Norartocarpetin (**13**) and morusin (**15**) exhibited modest protection effects with about 20% cell viability. Oxyresveratrol (**6**) and sanggenon C (**16**) at the concentration of 10 μM attenuated glutamate-induced toxicity weakly (about 12% cell viability), similar to resveratrol. It has been reported that moracin O (**5**) and moracin P (**8**) were promising drug candidates for cerebral ischemia and other neurodegenerative diseases due to their protective activities against oxygen-glucose deprivation (OGD)-induced cell death [40]. Therefore, we also tested the neuroprotective effects of compounds **1**–**5**, **7**–**9** against OGD-induced damage and 3-nitropropionic acid (3-NP)-induced neuronal injury on human neuroblastoma SH-SY5Y cells using the MTT method. Our results demonstrated that only moracin O (**5**), P (**8**), and moracin P 3′-*O*-β-d-glucopyranoside (**3**) indicated weak neuroprotective activities against OGD-induced damage, and none of them protected cells from the 3-NP-induced damage (Table 3).

### 2.3. Postulating the Neuroprotective Pathway by Molecular Docking

In order to further understand the possible mechanisms of the benzofuran stilbenes against glutamate-induced neuronal death, 10 glutamate receptors, including various ionotropic and metabotropic types, were selected from 64 *Homo sapiens* GluRs in the Protein Data Bank (PDB) website to study the ligand binding to these receptors in a molecular-docking method.

Among the selected glutamate receptors, the mGluR_1_ (3KS9) was the only one that could be docked with all active compounds (**5**–**8**, **13**, and **15**) as well as glutamic acid. In contrast, all of the inactive compounds (**1**–**4**, **9**) could not be docked into the binding site of the mGluR_1_ (3KS9). These results indicated that the neuroprotective activities of moracin O (**5**), R (**7**), and P (**8**), might be mediated by the mGluR_1_ pathway.

**Figure 3:**
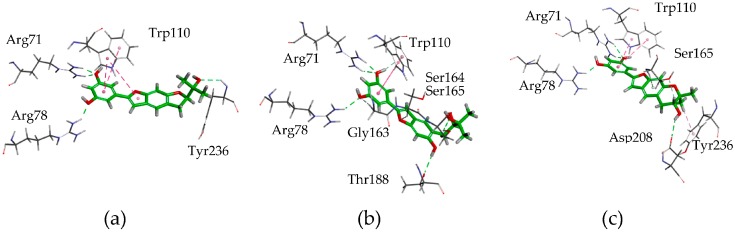
Binding models of moracin O (**5**), R (**7**), P (**8**) with the key residues of metabotropic glutamate receptor 1 (mGluR_1_) (3KS9). (**a**) Moracin O; (**b**) Moracin R; (**c**) Moracin P.

Molecular-docking results (Figure 3) showed each of the three ligands, moracin O (**5**), R (**7**), and P (**8**), had π–π interaction between the aromatic plane and Trp 110, and three to four H-bonds between hydroxyl groups of the ligands and the residues of Arg 71, Arg 78, Tyr 236 (or Asp 208, Ser165, Thr188) in mGluR_1_, which indicated that the aromatic group and the hydroxyl group play an important role in the bindings.

### 2.4. Analgesic Activities

Although some compounds from the genus *Morus* exhibited promising neuroprotective activity, *Morus* species have been rarely used to treat neurodegenerative diseases or other CNS diseases. As mGlu receptors had been considered to be valid targets for chronic pain control due to their ability of modulating rather than mediating excitatory synaptic activity [41], it was reasonable to hypothesize that these neuroprotective isoprenylated stilbenes might be the active analgesic components of Cortex Mori Radicis*.*

The three most effective neuroprotective stilbenes—moracin O (**5**), R (**7**), P (**8**)—and reported [20] flavonoid morusin (**15**) were assayed with the analgesic test and paracetamol was a positive control. As predicted, both moracin O (**5**) and P (**8**), with cyclic structural moieties, demonstrated more than 95% of inhibitions of acetic acid-induced pain at 80 mg/kg, which were as effective as morusin, much better than paracetamol. These results were consistent with the reported inhibition of morusin, which was about 73% at 60 mg/kg [20]. However, moracin R (**7**), which had an acyclic moiety, showed moderate inhibition (Table 4).

As compounds **1**–**4** and **9** were the glycosides of moracin O (**5**) and P (**8**), they might act as predrugs through oral administration, although they did not show neuroprotective effects in vitro.

## 3. Materials and Methods

### 3.1. Instruments

Melting points were determined on an XT5B melting point apparatus (Beijing Keyi Electric Light Instrument Factory, Beijing, China) and were uncorrected. Optical rotations were measured with a P-2000 polarimeter (Jasco, Tokyo, Japan). ECD spectra were recorded at room temperature with a J-815 spectropolarimeter (Jasco). UV spectra were collected in MeOH on a V-650 spectrophotometer (Jasco). IR spectra were recorded on a Nicolet 5700 spectrometer (Thermo, Madison, WI, USA) by the FT-IR transmission electron microscopy method. ^1^H- and ^13^C-NMR spectra were acquired using a DD2-500 spectrometer (Agilent, Santa Clara, CA, USA) or an AVIIIHD 600 spectrometer (Bruker, Billerica, MA, USA). HRESIMS were recorded on a 1200 series LC/6520 quadrupole time of flight (QTOF) spectrometer (Agilent). Column chromatography (CC) purification was performed using silica gel (160–200 mesh), Sephadex LH-20 (GE, Boston, MA, USA) and C_18_ (50 μm, YMC, Kyoto, Japan). CC fractions were analyzed by thin-layer chromatography (TLC) (silica gel GF_254_).

### 3.2. Plant Material

The Cortex Mori Radicis were bought from Anguo herb market, Hebei, China, which were collected from Hunan Province, China, in 2012. These samples were identified by Professor Lin Ma, Institute of Materia Medica, Chinese Academy of Medical Science and Peking Union Medical College, China. A voucher specimen (ID-S-2604) is deposited in the Institute of Materia Medica, Chinese Academy of Medical Science and Peking Union Medical College, China. The HPLC (high-performance liquid chromatography) profile of this plant material is in the Supplementary Materials. 

### 3.3. Animals

Male ICR (Institute of Cancer Research) mice (18–22 g) from Vital River Laboratories (Beijing, China) were used in these experiments. The animals were housed in a soundproof room at an appropriate temperature (25 ± 1 °C) and humidity (50% ± 10%) for three days before the experiment. They were kept under a 12 h light/12 h dark cycle (light from 7:00 a.m. to 7:00 p.m.) with free access to food and water. Every effort was made to minimize the number of experimental animals and the discomfort that they might experience. All animals were treated humanely in compliance with the “Principles of Laboratory Animal Care” and the “Guide for the Care and Use of Laboratory Animals of Peking Union Medical College and Chinese Academy of Medical Sciences”. All experimental protocols were approved by the Animal Care and Use Committee of the College (Approval NO. 00000251).

### 3.4. Extraction and Isolation

Powdered Cortex Mori Radicis (50 kg) were soaked with EtOH/H_2_O (50:50, *v/v*) for 24 h and percolated with 300 L EtOH/H_2_O (50:50, *v/v*). Then, evaporation of the solvent under reduced pressure gave a liquid extract, which was suspended in H_2_O and partitioned with EtOAc. The EtOAc extract (ca. 1 kg) was applied to a silica gel column (160–200 mesh, 2 kg), with a gradient elution (CHCl_3_/MeOH = 1:0–1:1, *v/v*) to yield fractions D1–D22. Fraction D7 and D8 was subjected to a silica gel column and eluted with an eluent (CHCl_3_/acetone = 1:0–1:1, *v/v*) to give 15 subfractions. The fifth subfraction was purified by a Sephadex LH-20 column, eluted with MeOH, to yield compound **15** (32 mg). The 8–12th subfraction was separated by MCI gel using a mobile phase of MeOH–H_2_O and followed by preparative HPLC to afford compound **5** (25 mg), **7** (20 mg), **8** (53 mg), **13** (5 mg), **16** (15 mg), **17** (8 mg), and **18** (5 mg). Fraction D11 and D12 was applied to RP C_18_ column, this being eluted with an eluent (MeOH/H_2_O) to give 11 subfractions. Compound **6** (2.3 g) was precipitated from the third subfraction. The fourth subfraction was purified by preparative HPLC to yield compound **14** (320 mg). Fraction D13–D15 was applied to a Sephadex LH-20 column, using MeOH/H_2_O as eluent, to give eight subfractions. The third subfraction was purified by a Sephadex LH-20 column, eluted MeOH, and then purified by HPLC to yield compound **1** (7 mg), **2** (3 mg), **3** (2 mg), and **4** (4 mg). The fifth subfraction was purified by a Sephadex LH-20 column and preparative HPLC, to yield compound **10** (2 mg), **11** (3 mg), and **12** (3 mg). The seventh subfraction was subjected to a silica gel column and eluted by petroleum ether/EtOAc (1:0–1:1) to yield compound **9** (10 mg). The retention times of all isolated compounds were determined by the HPLC profile in Supplementary Materials.

*Moracin P 3'-O-α-l-Arabinopyranoside* (**1**). Brown powder, m.p. 135.6–137.4 °C; $[\alpha {]}_{D}^{20}$ −65.9 (*c* 0.1, MeOH); UV (MeOH) λ_max_ (log ε) 219, 320 (3.54), 333 nm; CD (*c* 0.25, MeOH) [θ]_237.5_ −8440, [θ]_307_ −3409, [θ]_329.5_ −2380; IR ν_max_ 3387, 2979, 2926, 1605, 1577, 1461, 1352, 1173, 1146, 1108, 1176, 947, 859, 799 cm^−^^1^; ^1^H- and ^13^C-NMR data, Table 1 and Table 2; HRESIMS *m/z* 459.1663 [M + H]^+^ (calcd for C_24_H_27_O_9_^+^, 459.1650).

*Moracin P 9-O-β-d-Glucopyranoside* (**2**). Brown powder, m.p. 123.3–124.4 °C; $[\alpha {]}_{D}^{20}$ −183.4 (*c* 0.1, MeOH); UV (MeOH) λ_max_ (log ε) 219, 319 (3.34), 333 nm; CD (*c* 0.25, MeOH) [θ]_236.5_ −4724, [θ]_324_ −1815; IR ν_max_ 3358, 2977, 2930, 1606, 1579.7, 1461.9, 1354, 1150, 1107, 1080, 1036, 947, 845 cm^−^^1^; ^1^H- and ^13^C-NMR, Table 1 and Table 2; HRESIMS *m/z* 511.1588 [M + Na]^+^ (calcd for C_25_H_28_O_10_Na^+^, 511.1575).

*Moracin P 3'-O-β-d-Glucopyranoside* (**3**). Brown powder, $[\alpha {]}_{D}^{20}$ −47.6 (*c* 0.1, MeOH); m.p. 136.3–137.2 °C; UV (MeOH) λ_max_ (log ε) 219, 321 (3.28), 334 nm; CD (*c* 0.25, MeOH) [θ]_234_ −3966, [θ]_317.5_ −2390; IR ν_max_ 3341, 2977, 2927, 1619.6, 1576.6, 1461.8, 1352, 1174, 1142, 1078, 1023, 996, 850 cm^−^^1^; ^1^H- and ^13^C-NMR data, Table 1 and Table 2; HRESIMS *m/z* 511.1588 [M + Na]^+^ (calcd for C_25_H_28_O_10_Na^+^, 511.1575).

*Moracin O 10-O-β-d-Glucopyranoside* (**4**). Brown powder, m.p. 119.5–120.5 °C; $[\alpha {]}_{D}^{20}$ −148.3 (*c* 0.1, MeOH); UV (MeOH) λ_max_ (log ε) 219, 322 (3.36), 336 nm; CD (*c* 0.25, MeOH) [θ]_237_ −6551, [θ]_315_ −1607; IR ν_max_ 3349, 2979, 2922, 1619, 1578.8, 1456.8, 1359, 1163, 1084, 1042, 951, 876, 845 cm^−^^1^; ^1^H- and ^13^C-NMR data, Table 1 and Table 2; HRESIMS *m/z* 511.1589 [M + Na]^+^ (calcd for C_25_H_28_O_10_Na^+^, 511.1575).

### 3.5. Acid Hydrolysis of Compounds ***1**–**4***

Compounds **1**–**4** (ca. 0.5 mg) were dissolved in 0.5 N HCl (0.1 mL) and heated at 90 °C for 2 h. The solution was neutralized with aqueous ammonia and partitioned with EtOAc (0.2 mL × 3). After drying the H_2_O-soluble fraction in vacuo, the residue was dissolved in pyridine (0.1 mL) containing l-cysteine methyl ester hydrochloride (0.5 mg) and heated at 60 °C for 1 h. A 0.1 mL solution of *o*-torylisothiocyanate (0.5 mg) in pyridine was added to the mixture, which was heated at 60 °C for 1 h. The reaction mixture was directly analyzed by analytical HPLC under the following conditions: column was Grace Prevail C_18_ (4.6 × 250 mm, 5 μm, Grace, Deerfield, IL, USA); the mobile phase was acetonitrile and 50 mM H_3_PO_4_ (25:75); flow rate was 0.8 mL/min; wavelength was 250 nm; column temperature was 35 °C. Under the above conditions, authentic d/l-glucose, d/l-arabinose gave HPLC peaks at *t*_R_ 20.6/18.8 and 24.0/22.3 min, respectively. The *t*_R_ of the sugars of the isolates obtained by acid hydrolysis gave similar results as those of standard sugars.

### 3.6. Neuroprotective Assay

Human neuroblastoma SH-SY5Y cells were grown in Dulbecco’s Modified Eagle’s Medium (DMEM) (Sigma-Aldrich, St. Louis, MO, USA) containing 10% fetal bovine serum (FBS), 100 U/mL penicillin/streptomycin. Cell cultures were incubated at 37 °C in a humid 5% CO_2_/95% air environment. SK-N-SH cells were cultured in 96-well microplates at a density of 1 × 10^5^ cells/well. Compounds were prepared in DMSO as 100 mM stock solution. Glutamate and 3-NP were freshly prepared prior to each experiment. Na_2_S_2_O_4_ was prepared in phosphate buffer solution (PBS) as 50 mM stock solution, stored in a refrigerator at −20 °C and kept away from light. The cells were preincubated with compounds for 4 h, and then the cells were separately incubated with 30 mM glutamate for 4 h, 5 mM Na_2_S_2_O_4_ with DMEM lacking d-glucose solution for 24 h, and 10 μM 3-NP solution for 24 h. After the scheduled time, MTT solution (5 mg/mL) was added for another 4 h at 37 °C. MTT formazan crystals were solubilized by DMSO and spectrophotometrically measured at 570 nm (the max. emission wavelength of detected samples was at 410 nm). All data presented in our study were obtained from at least three independent experiments and expressed as the mean ± SEM (standard error of the mean). Significant differences between groups were compared using the one-way ANOVA procedure followed by a least significant difference (LSD) post hoc test using SPSS ver. 10.0 software (SPSS Inc., Chicago, IL, USA). The differences were considered statistically significant at *p* < 0.05.

### 3.7. Acetic Acid-Induced Abdominal Constrictions

ICR mice (*n* = 10 each group) were pretreated intraperitoneally (i.p.) with the compounds (80 mg/kg) in a separate set or standard drug paracetamol (200 mg/kg) i.p. 30 min before acetic acid injection (0.7%). Control animals received a similar volume of the appropriate vehicle (10 mL/kg) used to dilute the compounds.

The mice were placed individually into glass beakers and 5 min were allowed to elapse. The mice were then observed for a period of 10 min and the number of writhes was recorded for each animal. For scoring purposes, a writhe was indicated by stretching of the abdomen with simultaneous stretching of at least one hind limb. The formula for computing percent inhibition was: average writhes in the control group minus writhes in the drug group divided by writhes in the control group times 100%.

### 3.8. Molecular Modeling

Computational molecular modeling studies were carried out using Discovery Studio 4.1.0 (Accelrys, San Diego, CA, USA). All structures of glutamate receptors (2NZS, 1S50, 3UA8, 4F39, 4NF4, 3KS9, 5CNI, 5CNK, 5C5C, and 3LMK) were downloaded from the PDB website. The binding site was defined from the PDB site records. The screening of glutamate receptors was carried out by positioning the ligand in the binding site using CDocker [42], and evaluated by –CDocker energy. The interaction of ligand and receptor was further calculated using Flexible Docking [43] and the conformation of ligand and protein-binding site was evaluated by –CDocker interaction energy.

## 4. Conclusions

In summary, 4 new benzofuran-type stilbene glycosides (**1**–**4**) along with 14 known compounds were isolated from the traditional Chinese medicines, Cortex Mori Radicis. The new compounds were identified as moracin P glycosides and moracin O glycosides. Three isoprenylated benzofuran-type stilbenes, moracin O (**5**), R (**7**), and P (**8**), showed significant neuroprotective activity against glutamate-induced cell death in SK-N-SH cells. The molecular-docking results indicated that the active stilbenes could be mediated by the mGluR_1_ pathway for their neuroprotective activities. As glutamate receptors may be implicated in persistent or chronic pain, we hypothesize that these neuroprotective isoprenylated stilbenes might be the active analgesic components of the genus *Morus*, and this opinion was confirmed by the inhibition of the acetic acid-induced pain by three stilbenes. Although the neuroprotective effect on glutamate-induced neuron death of benzofuran-type stilbenes such as moracin E was reported [44], this paper firstly postulated their mGluR_1_ pathway and correlated the neuroprotective activity with analgesic activity, which was related with the traditional antirheumatic usage of *Morus* species. As compounds **1**–**4** and **9** were the glycosides of moracin O (**5**) and P (**8**), they may act as predrugs through oral administration, although they did not show neuroprotective effect in vitro.

As only two analgesic compounds were reported in *Morus* plants, we believe that our findings gave a new alternative method to discover and evaluate the analgesic components in *Morus* species.

## Figures and Tables

**Table 1:** ^1^H-NMR (500 MHz, *J* in Hz) spectroscopic data of compounds **1**–**4** in DMSO-*d*_6_.

No.	1	2	3	4
3	7.17 s	7.07 s	7.15 s	7.07 s
4	7.29 s	7.30 s	7.27 s	7.34 s
7	6.93 s	6.94 s	6.92 s	6.98 s
8	3.02 dd (16.4, 6.0), 2.72 dd (16.4, 7.0)	3.14 m ^b^, 2.83 dd (16.6, 6.7)	3.01 dd (16.5, 5.2), 2.70 dd (16.5, 8.1)	3.16 m ^c^, 3.35 m *
9	3.67 dd (7.0, 6.0)	3.95 dd (6.7, 5.9)	3.66 dd (8.1, 5.2)	4.75 t (8.8)
11	1.31 s	1.34 s	1.31 s	1.28 s
12	1.18 s	1.24 s	1.18 s	1.21 s
2′	6.91 d (2.1)	6.68 d (1.5)	6.96 br s	6.67 d (2.0)
4′	6.43 t (2.1)	6.21 t (1.5)	6.44 br s	6.21 t (2.0)
6′	6.89 d (2.1)	6.68 d (1.5)	6.88 br s	6.67 d (2.0)
1″	4.85 d (6.7)	4.33 d (7.7)	4.83 d (7.6)	4.42 d (7.8)
2″	3.60 m ^a^	2.94 dd (8.0, 7.7)	3.22 t (7.6)	2.88 t (8.2)
3″	3.49 dd (8.5, 3.2)	3.14 m ^b^	3.27 t (8.5)	3.16 m ^c^
4″	3.70 brs	3.03 t (9.1)	3.16 t (9.0)	3.04 t (8.0) ^d^
5″	3.60 dd (11.2, 1.5) ^a^, 3.75 dd (11.2, 3.2)	3.14 m ^b^	3.35 m *	3.04 d (8.0) ^d^
6″	----	3.68 br d (11.3), 3.43 dd (11.3, 6.2)	3.71 br.d (11.4), 3.48 m	3.35 m *, 3.45 br.d (11.3)

^a–d^ Signals were overlapped with each other. * Signals were overlapped by water or solvent peak.

**Table 2:** ^13^C-NMR spectroscopic data of compounds **1–4** in DMSO-*d*_6_.

Position	1 ^a^	2 ^b^	3	4	Position	1 ^a^	2 ^b^	3	4
2	154.0	153.8	154.1	154.2	1′	131.5	131.5	131.6	131.6
3	101.8	101.1	101.7	101.7	2′	103.4	102.4	103.5	102.2
3a	121.9	122.2	122.0	122.0	3′	158.8	158.8	159.1	158.8
4	120.9	121.0	120.9	116.1	4′	103.9	102.8	103.8	102.6
5	117.2	116.5	117.2	124.3	5′	158.8	158.8	158.8	158.8
6	151.2	150.8	151.2	158.1	6′	104.8	102.4	104.8	102.2
7	98.4	98.5	98.4	92.3	1″	100.8	100.2	100.8	97.4
7a	153.8	154.6	153.9	154.2	2″	72.4	73.8	73.3	73.5
8	31.2	27.6	31.3	29.8	3″	70.3	77.0	76.6	77.0
9	67.4	73.4	68.1	88.9	4″	68.0	70.3	69.7	70.1
10	77.3	76.3	77.3	77.1	5″	65.5	77.0	77.1	76.6
11	25.8	25.8	25.8	23.4	6″		61.4	60.7	60.9
12	20.3	21.3	20.4	21.8					

Measured at ^a^ 125 MHz or ^b^ 150 MHz.

**Table 3:** Neuroprotective effects of compounds (10 μM) from Cortex Mori Radicis against the injured neuron cells.

Compounds	Cell Viability (%) ^a^		
Injured reagent	l-Glu ^b^	Na_2_S_2_O_4_^c^	3-NP ^c^
Control	100	100	100
Injured control	0.0 ^###,d^	0.0 ^###,e^	0.0 ^###,f^
Moracin P 3’-*O*-α-l-arabinopyranoside (**1**)	−5.0 ± 2.6	−0.4 ± 1.3	----
Moracin P 9-*O*-β-d-glucopyranoside (**2**)	−2.0 ± 1.5	4.4 ± 1.34	----
Moracin P 3’-*O*-β-d-glucopyranoside (**3**)	1.9 ± 3.3	7.1 ± 2.52	----
Moracin O 10-*O*-β-d-glucopyranoside (**4**)	−4.4 ± 0.4	−1.4 ± 0.5	----
Moracin O (**5**)	56.0 ± 5.1 **	12.4 ± 1.5	----
Oxyresveratrol (**6**)	12.7 ± 5.4	----	----
Moracin R (**7**)	50.0 ± 4.5 **	0.9 ± 1.4	−3.3 ± 2.6
Moracin P (**8**)	40.1 ± 4.4 *	9.0 ± 2.1	0.3 ± 3.8
Mulberroside C (**9**)	−6.0 ± 2.0	0.0 ± 2.1	----
Norartocarpetin (**13**)	21.7 ± 3.9	----	----
Morusin (**15**)	20.2 ± 4.8	----	----
Sanggenon C (**16**)	13.5 ± 4.4	----	----
Resveratrol ^g^	12.0 ± 2.4	----	----

^a^ The cell viability was calculated as 100 × (tested group − damaged group)/(control group − damaged group). The cell line was ^b^ SK-N-SH or ^c^ SH-SY5Y. The cell survival percentage was ^d^ 58.4 ± 3.1%, ^e^ 67.8% ± 1.5%, ^f^ 60.1% ± 1.8% compared with the control group. ^g^ Acted as positive control only in the glutamate-induced cell death assay. ^###^ *p* < 0.001 versus control group; * *p* < 0.05, ** *p* < 0.01 versus injured control group.

**Table 4:** Analgesic effects of neuroprotective stilbenes on acetic acid-induced pain.

Samples	Dosage (mg/kg, i.p.)	Mean Numbers of Writhes ± SD	Inhibition Ratio (%)
Control	0	17 ± 6	----
Moracin O (**5**)	80	0 ± 1 *	98%
Moracin R (**7**)	80	5 ± 3 *	76%
Moracin P (**8**)	80	1 ± 1 *	95%
Morusin (**15**)	80	1 ± 1 *	95%
Paracetamol	200	1 ± 1 *	95%

* *p* < 0.01 versus control group.

## Supplementary Materials

The Supplementary materials (copies of MS, UV, ECD, IR, and NMR spectra of compounds **1**−**4**, calculated ECD of **1a**, and HPLC profile of plant material) are available online at: http://www.mdpi.com/1420-3049/22/2/236/s1.
