# Supplementary material for: Bioactive Benzofuran Derivatives from Cortex Mori Radicis, and Their Neuroprotective and Analgesic Activities Mediated by mGluR1
Source: Molecules. 2017 Feb 8;22(2):236. doi: 10.3390/molecules22020236 (PMC6155743; doi:10.3390/molecules22020236)
Supplement: Supplementary file 1 [file molecules-22-00236-s001.pdf]

Supplementary Materials: Bioactive Stilbenes from Cortex Mori Radicis, and Their Neuroprotective and Analgesic Activities Mediated by mGluR1

Ya-Nan Wang, Mao-Feng Liu, Wei-Zhen Hou, Rui-Ming Xu, Jie Gao, An-Qi Lu, Mei-Ping Xie, Lan Li, Jian-Jun Zhang, Ying Peng, Li-Li Ma, Xiao-Liang Wang, Jian-Gong Shi and Su-Juan Wang

Supporting information

Figure SI-1a. ^{1}HNMR spectra of compound **1**
Figure SI-1b. ^{13}CNMR spectra of compound **1**
Figure SI-1c. HMQC spectra of compound **1**
Figure SI-1d. HMBC spectra of compound **1**
Figure SI-1e. (+)ESIMS spectra of compound **1**
Figure SI-1f. IR spectra of compound **1**
Figure SI-1g. CD and UV spectrum of compound **1**
Figure SI-1h. The absolute configuration of sugar in **1** determined by HPLC
Figure SI-2a. ^{1}HNMR spectra of compound **2**
Figure SI-2b. ^{13}CNMR spectra of compound **2**
Figure SI-2c. HMQC spectra of compound **2**
Figure SI-2d. HMBC spectra of compound **2**
Figure SI-2e. (+)ESIMS spectra of compound **2**
Figure SI-2f. IR spectra of compound **2**
Figure SI-2g. CD and UV spectrum of compound **2**
Figure SI-2h. The absolute configuration of sugar in **2** determined by HPLC
Figure SI-3a. ^{1}HNMR spectra of compound **3**
Figure SI-3b. ^{13}CNMR spectra of compound **3**
Figure SI-3c. HMQC spectra of compound **3**
Figure SI-3d. HMBC spectra of compound **3**
Figure SI-3e. (+)ESIMS spectra of compound **3**
Figure SI-3f. IR spectra of compound **3**
Figure SI-3g. CD and UV spectrum of compound **3**
Figure SI-3h. The absolute configuration of sugar in **3** determined by HPLC
Figure SI-4a. ^{1}HNMR spectra of compound **4**
Figure SI-4b. ^{13}CNMR spectra of compound **4**
Figure SI-4c. HMQC spectra of compound **4**
Figure SI-4d. HMBC spectra of compound **4**
Figure SI-4e. (+)ESIMS spectra of compound **4**
Figure SI-4f. IR spectra of compound **4**
Figure SI-4g. CD and UV spectrum of compound **4**
Figure SI-4h. The absolute configuration of sugar in **4** determined by HPLC
Table SI-1. Optical rotation of aglycones **1a**–**4a**, moracin O (**5**), and P (**8**)
Calculated ECD of **1a**
Figure SI-5a. Calculated ECD spectra of **1a**-C1–C3

Table SI-2. The Boltzmann distribution of three conformations (C1–C3) of **1a**
Figure SI-5b. MO related with two cotton effects of **1a**
HPLC profile of Cortex Mori Radicis and their main components identified by HPLC-MS
Figure SI-6. HPLC profile of Cortex Mori Radicis
Table SI-3. The main components of Cortex Mori Radicis identified by HPLC-MS

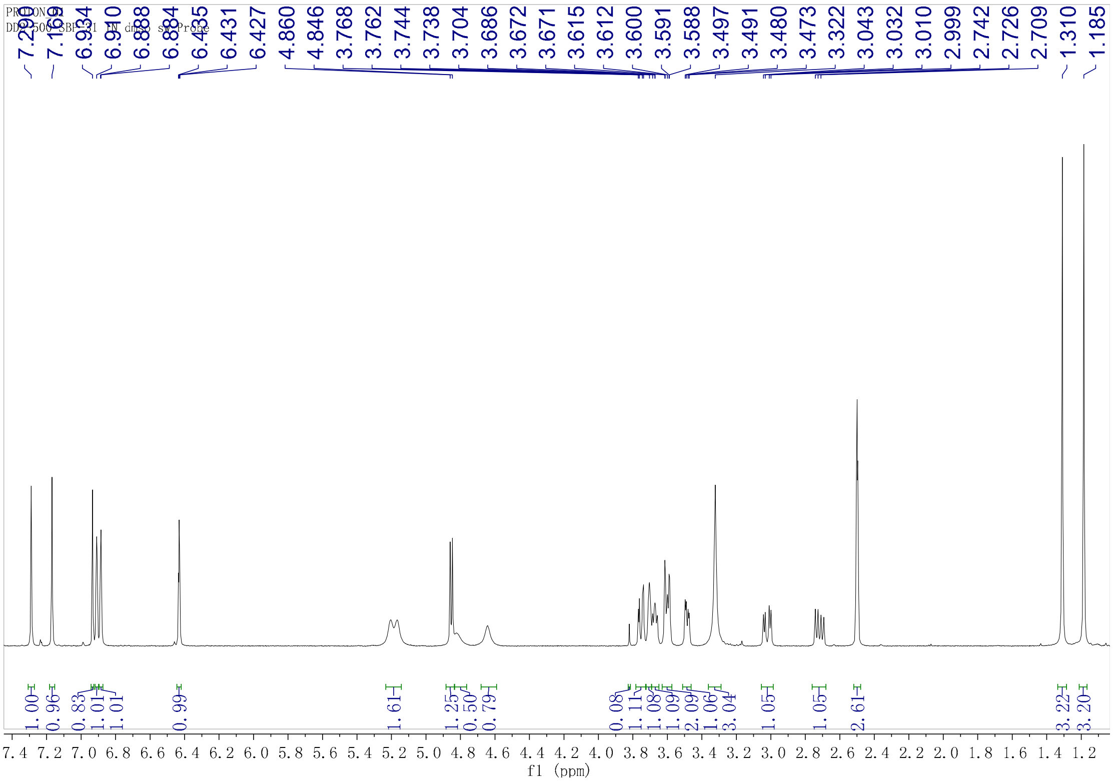

Figure SI-1a. ^{1}H NMR spectra of compound **1**.

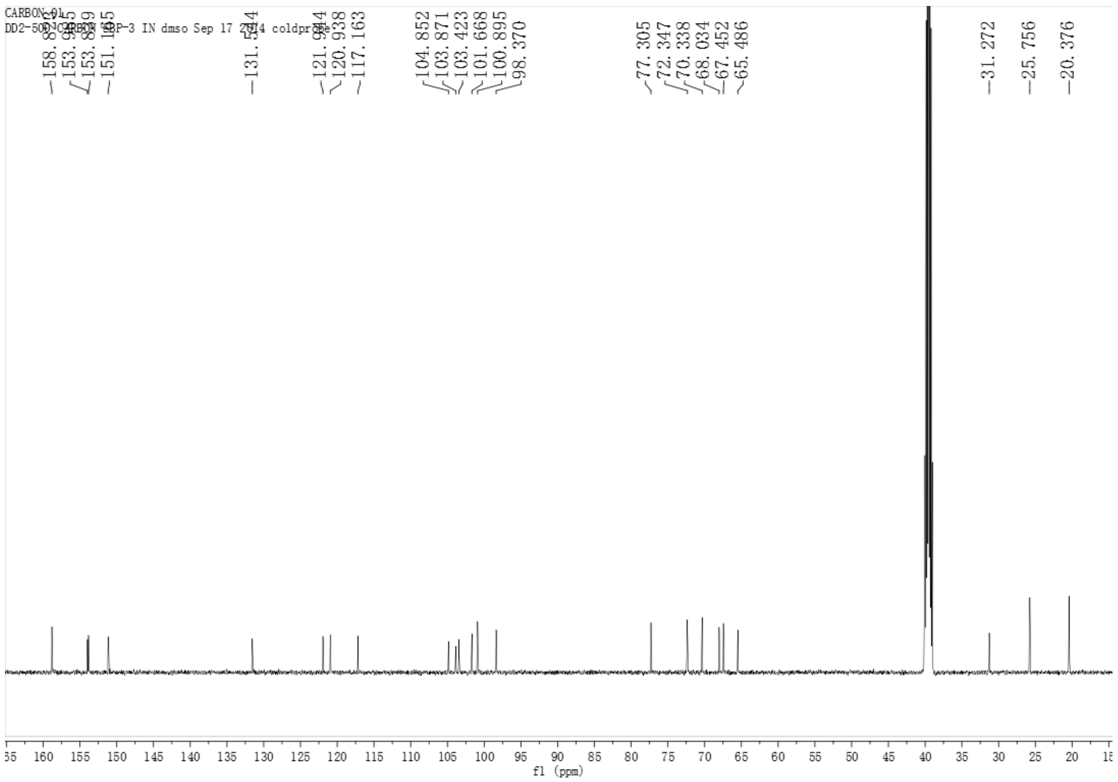

Figure SI-1b. ^{13}C NMR spectra of compound **1**.

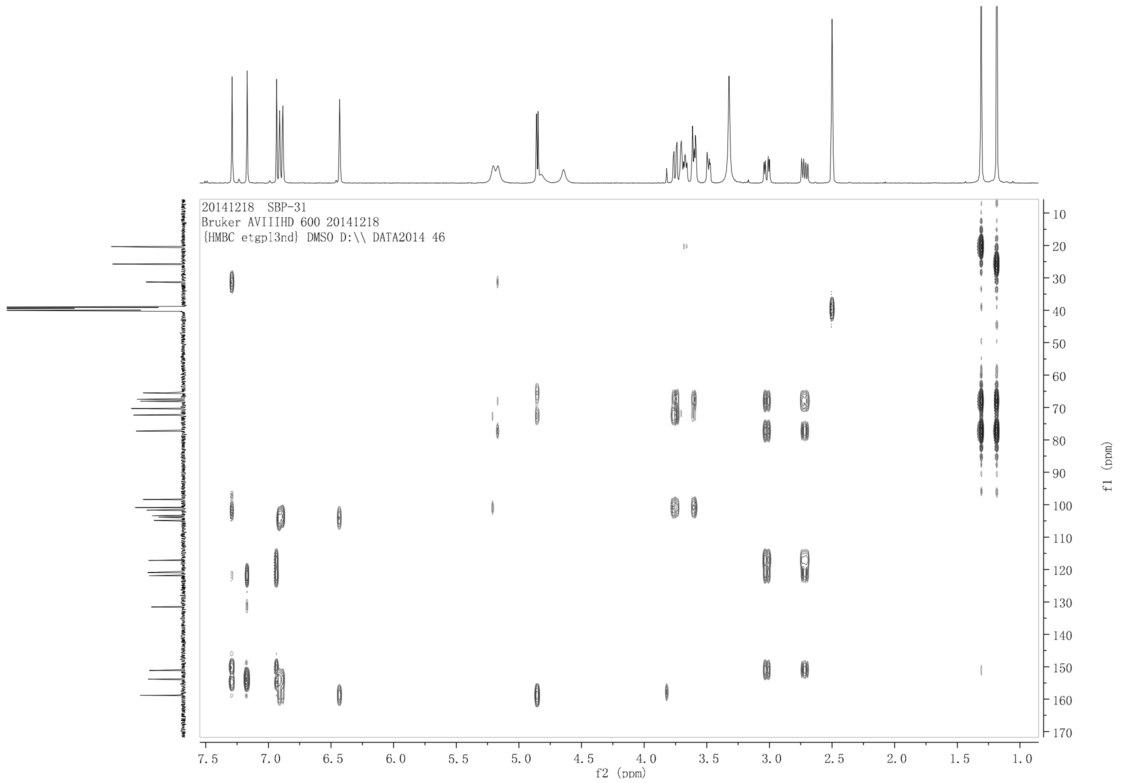

Figure SI-1c. HMQC spectra of compound **1**.

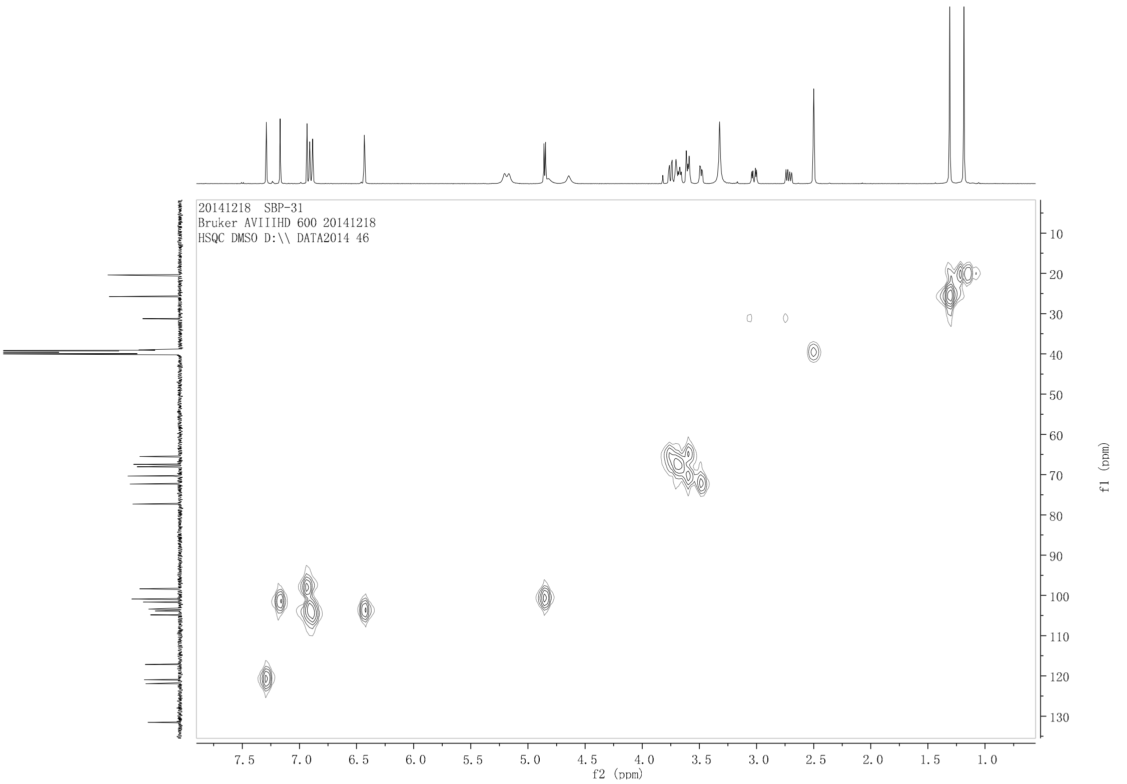

Figure SI-1d. HMBC spectra of compound **1**.

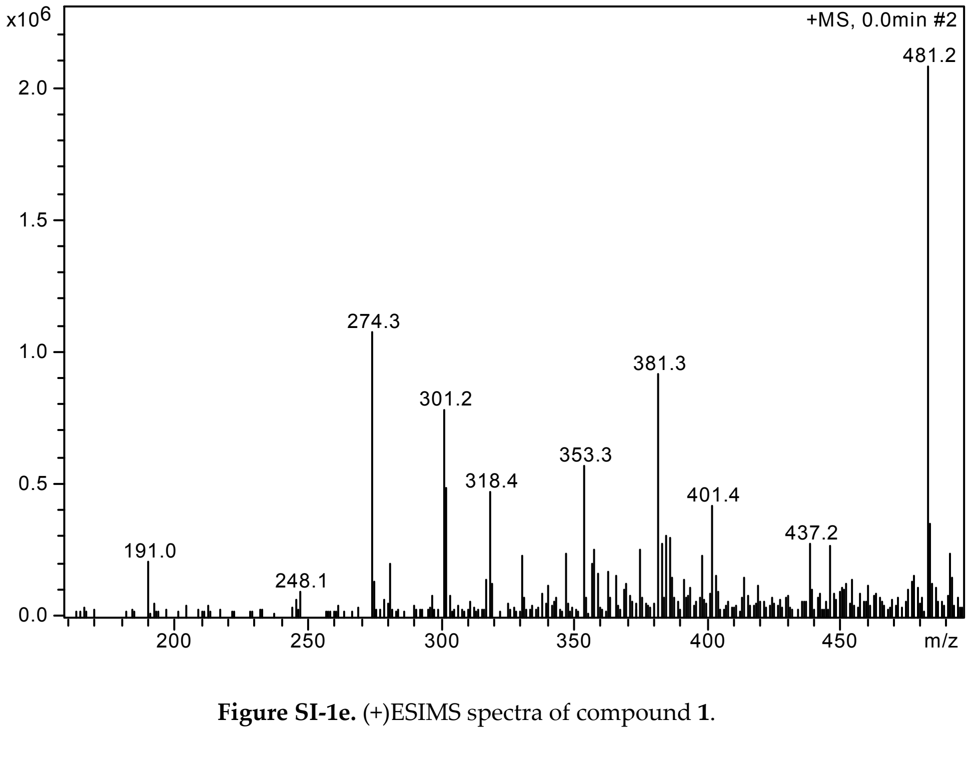

Figure SI-1e. (+)ESIMS spectra of compound **1**.

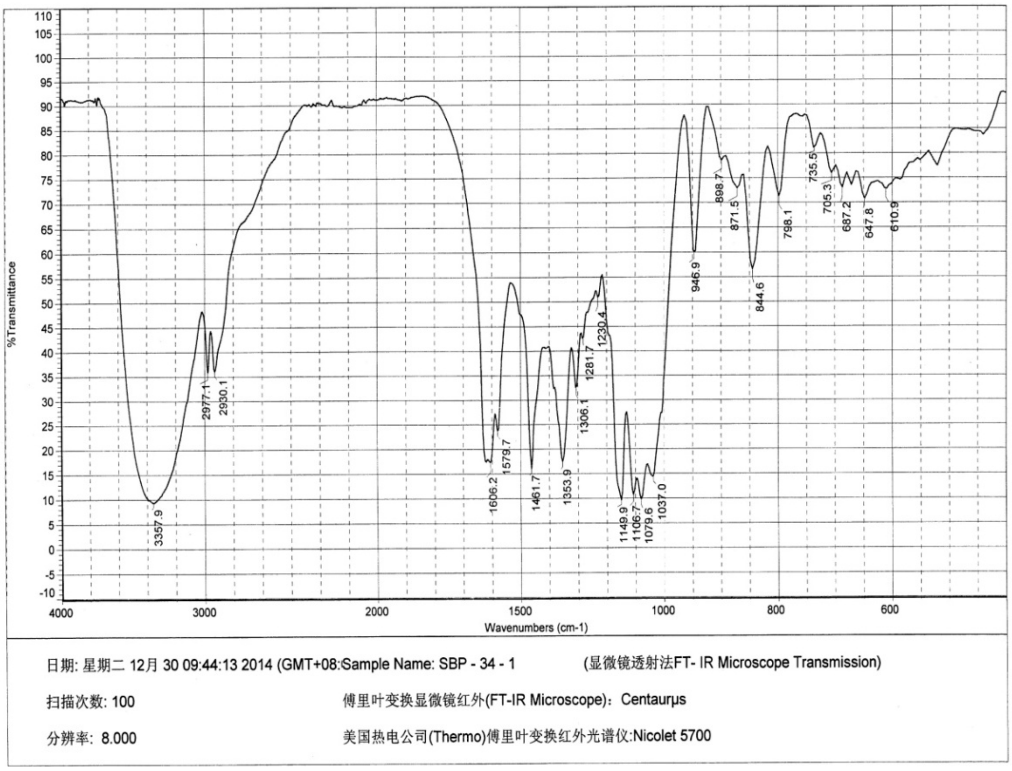

Figure SI-1f. IR spectra of compound **1**.

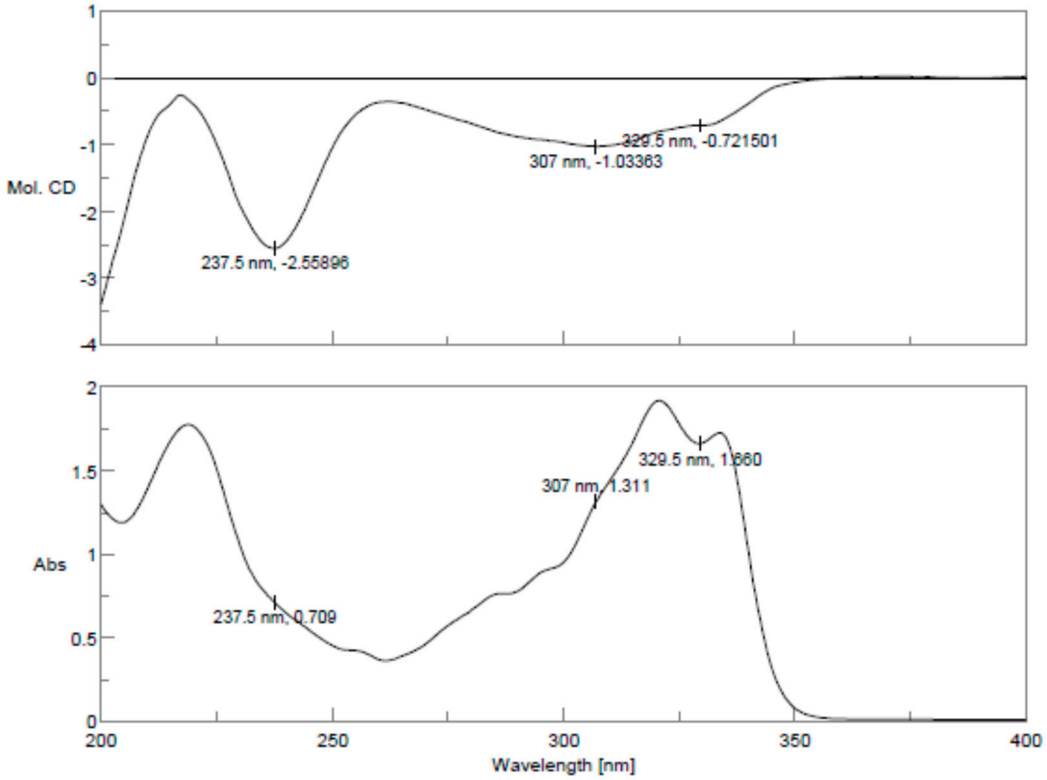

Figure SI-1g. CD and UV spectrum of compound **1**.

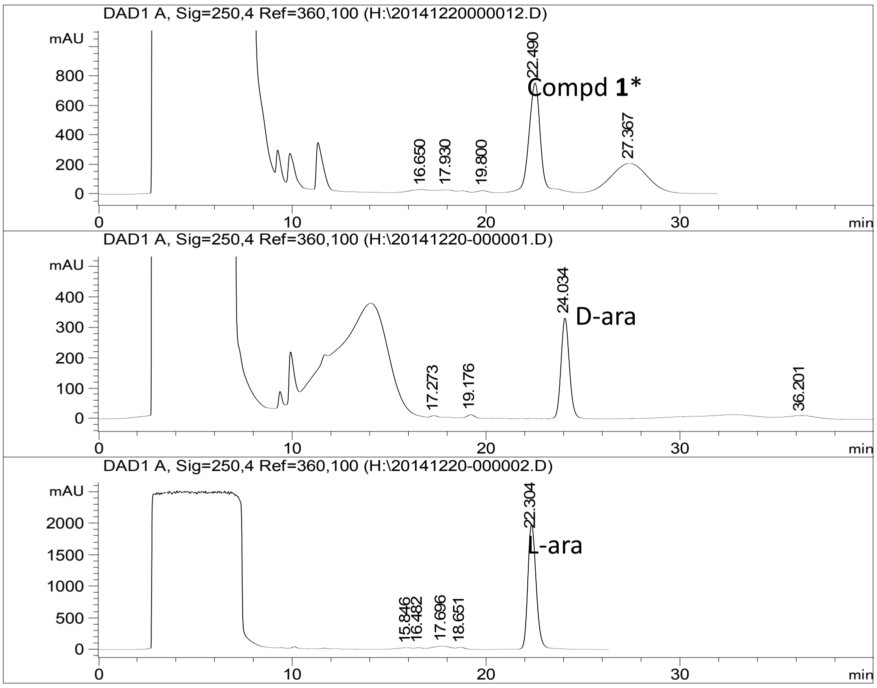

Figure SI-1h. HPLC of compound **1**, D-arabinose, L-arabinose.

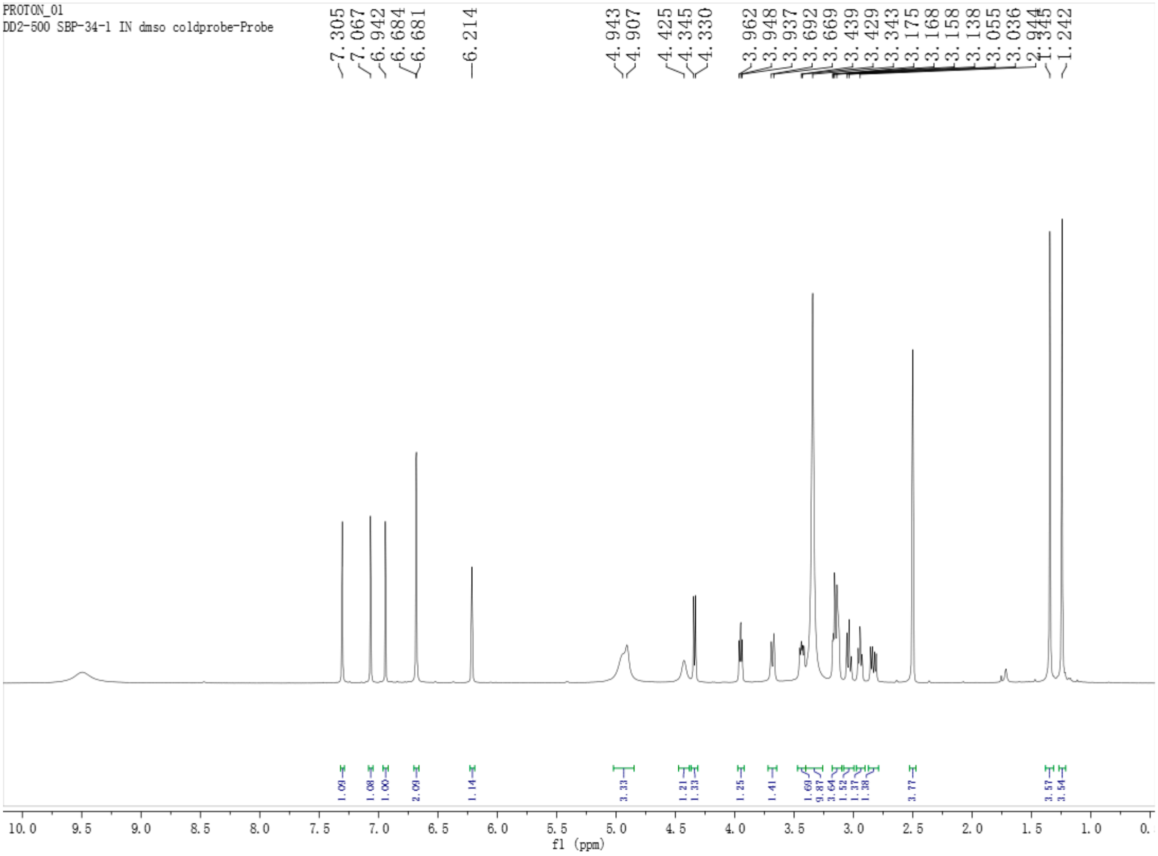

Figure SI-2a. ^{1}H NMR spectra of compound **2**.

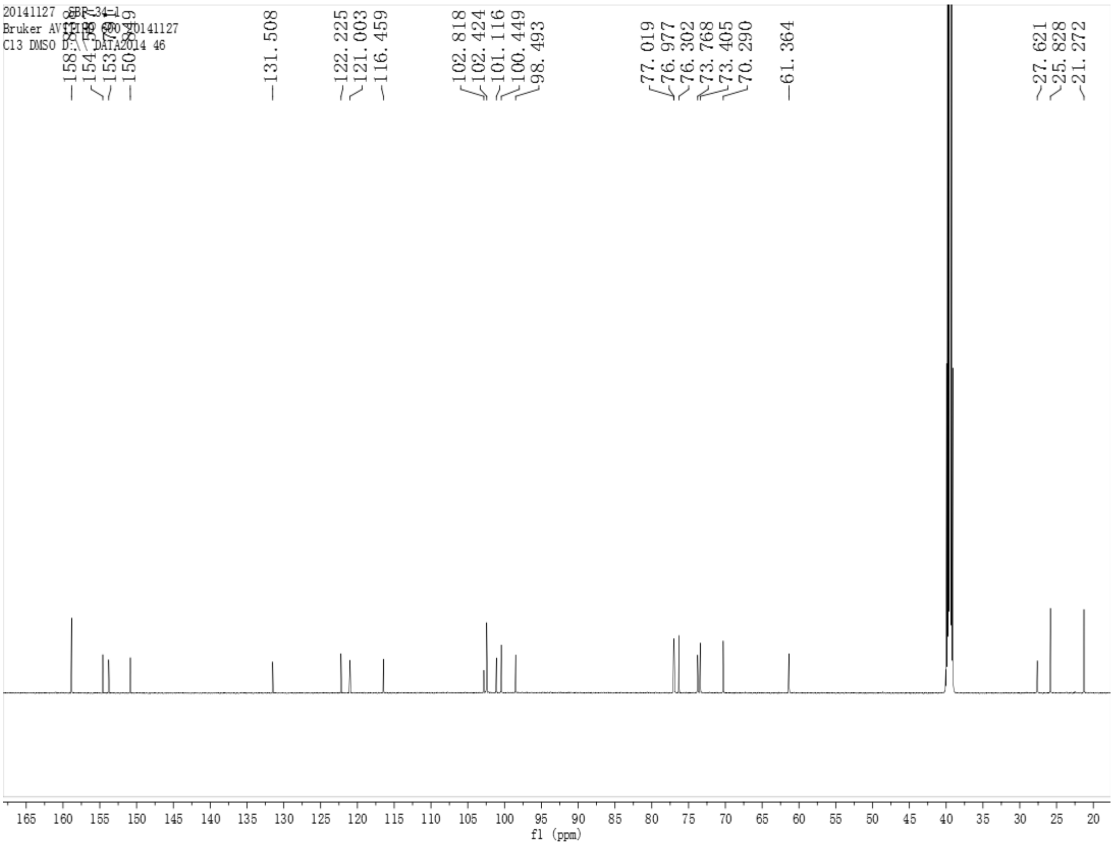

Figure SI-2b. ^{13}C NMR spectra of compound **2**.

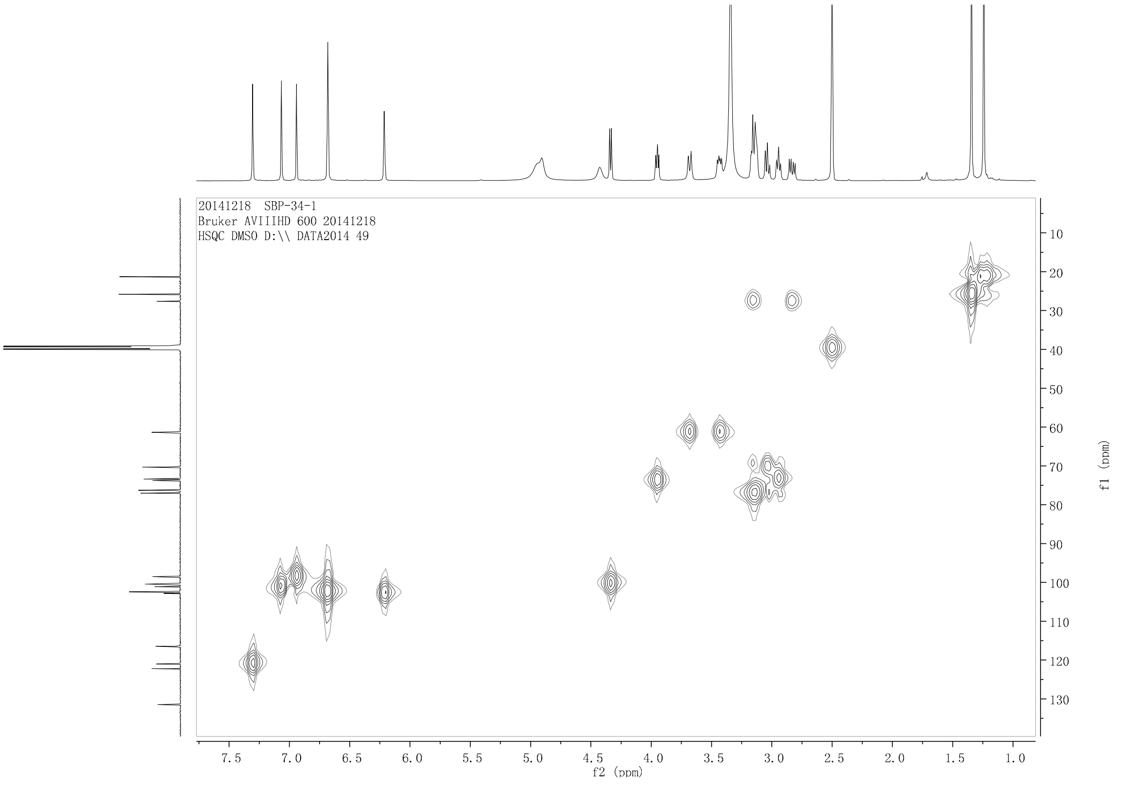

Figure SI-2c. HMQC spectra of compound **2**.

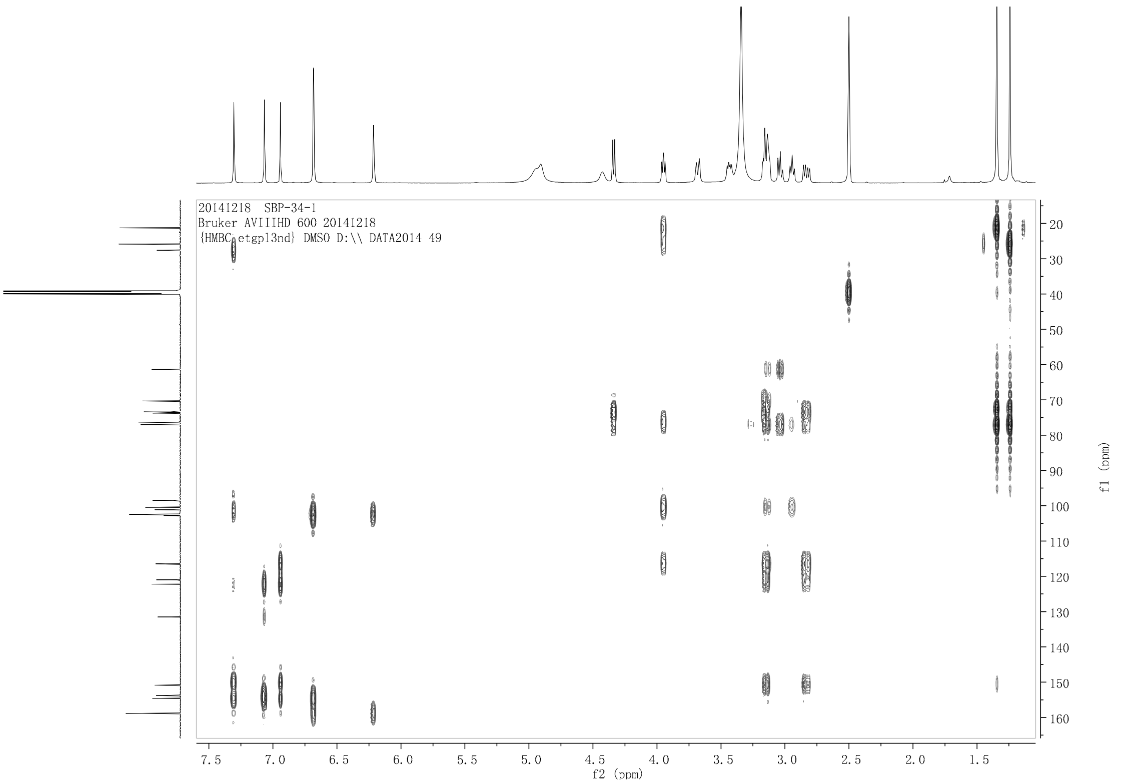

Figure SI-2d. HMBC spectra of compound **2**.

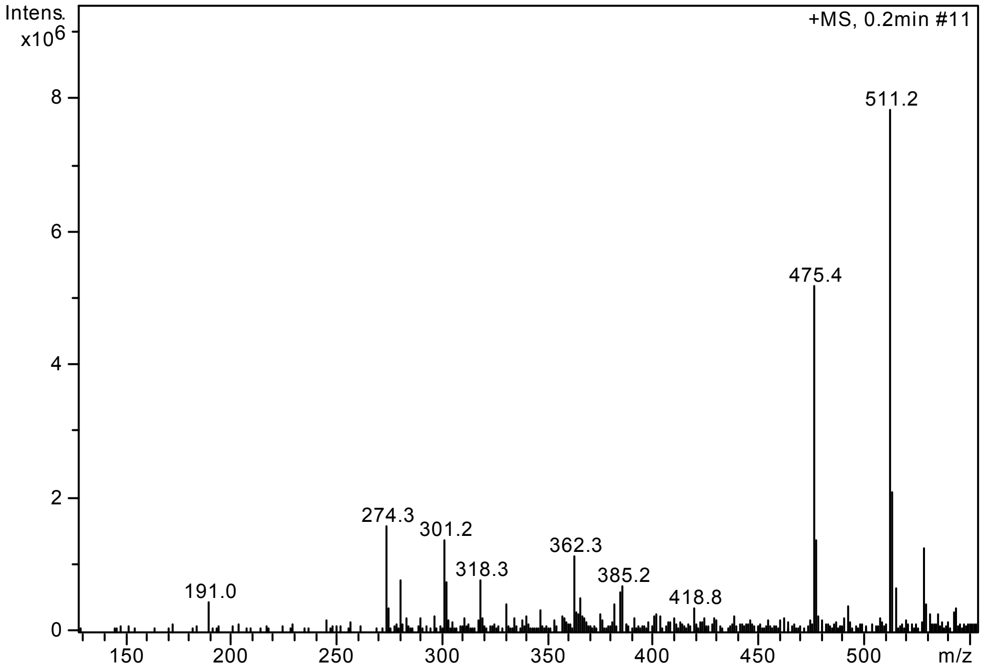

Figure SI-2e. (+)ESIMS spectra of compound **2**.

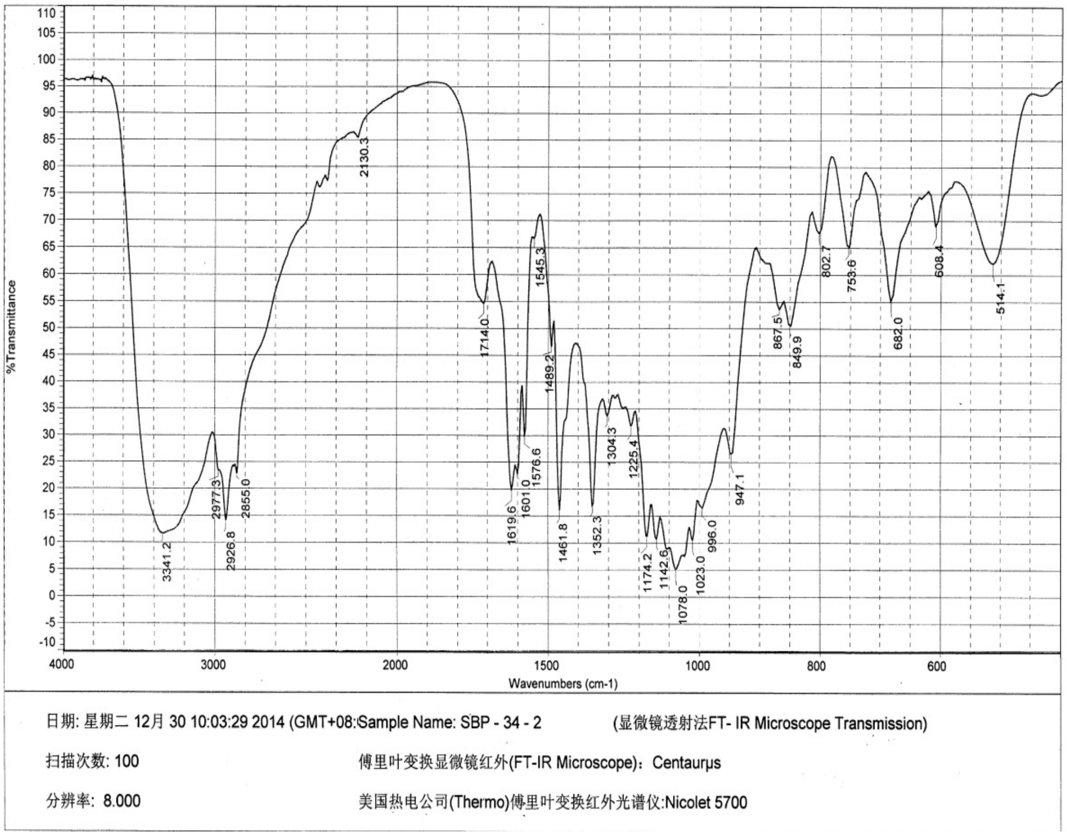

Figure SI-2f. IR spectra of compound **2**.

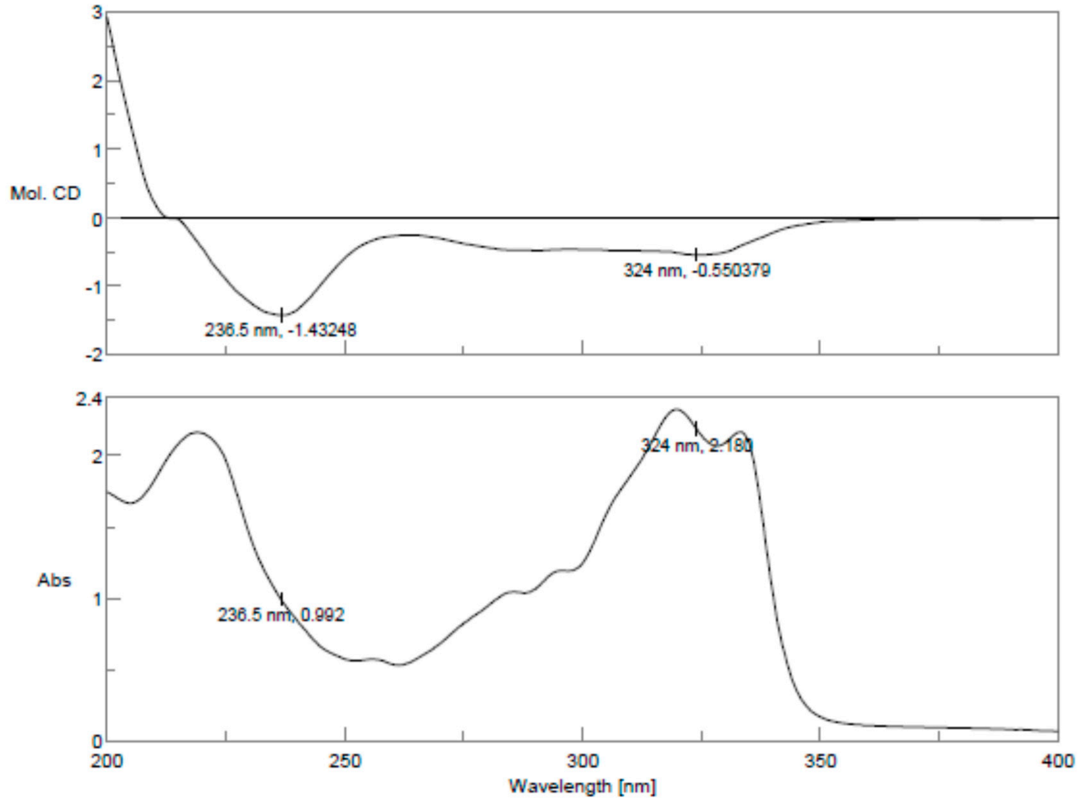

Figure SI-2g. CD and UV spectrum of compound **2**.

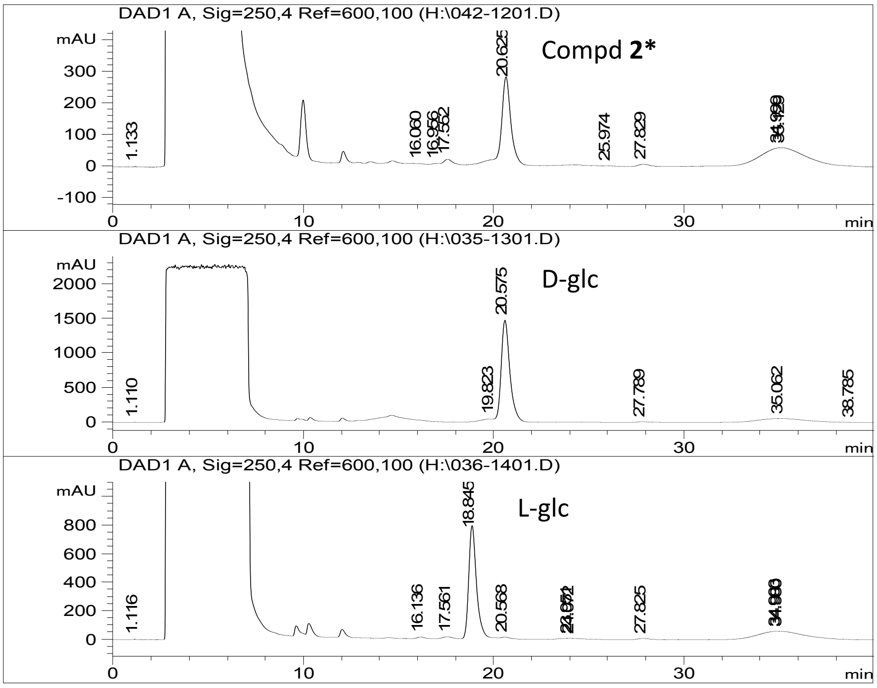

Figure SI-2h. HPLC of compound **2**, D-glucose and L-glucose.

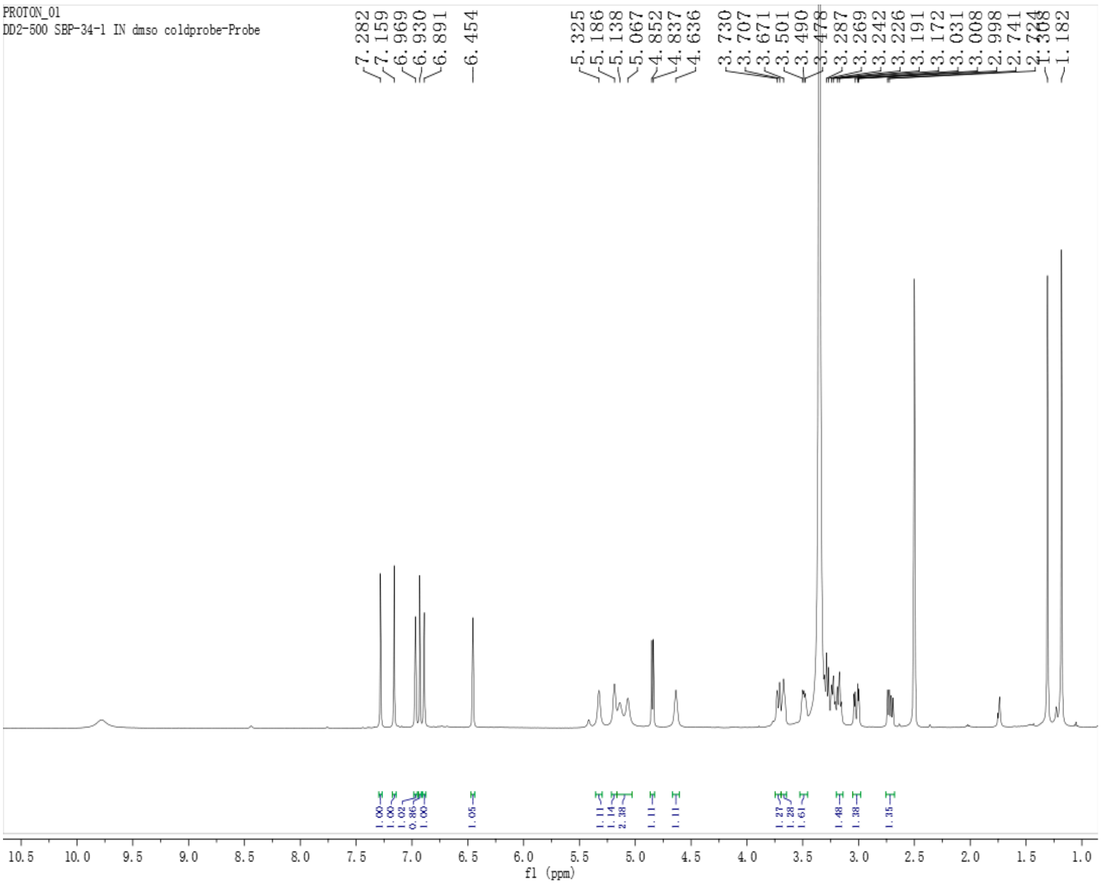

Figure SI-3a. ^{1}H NMR spectra of compound **3**.

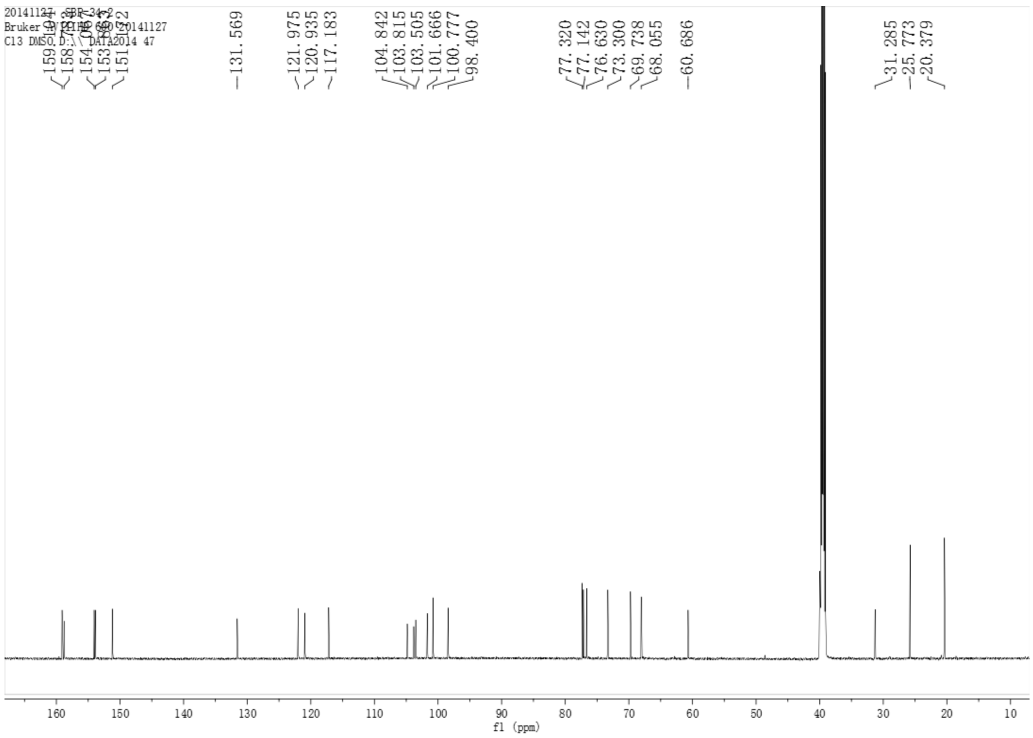

Figure SI-3b.^{13}C NMR spectra of compound **3**.

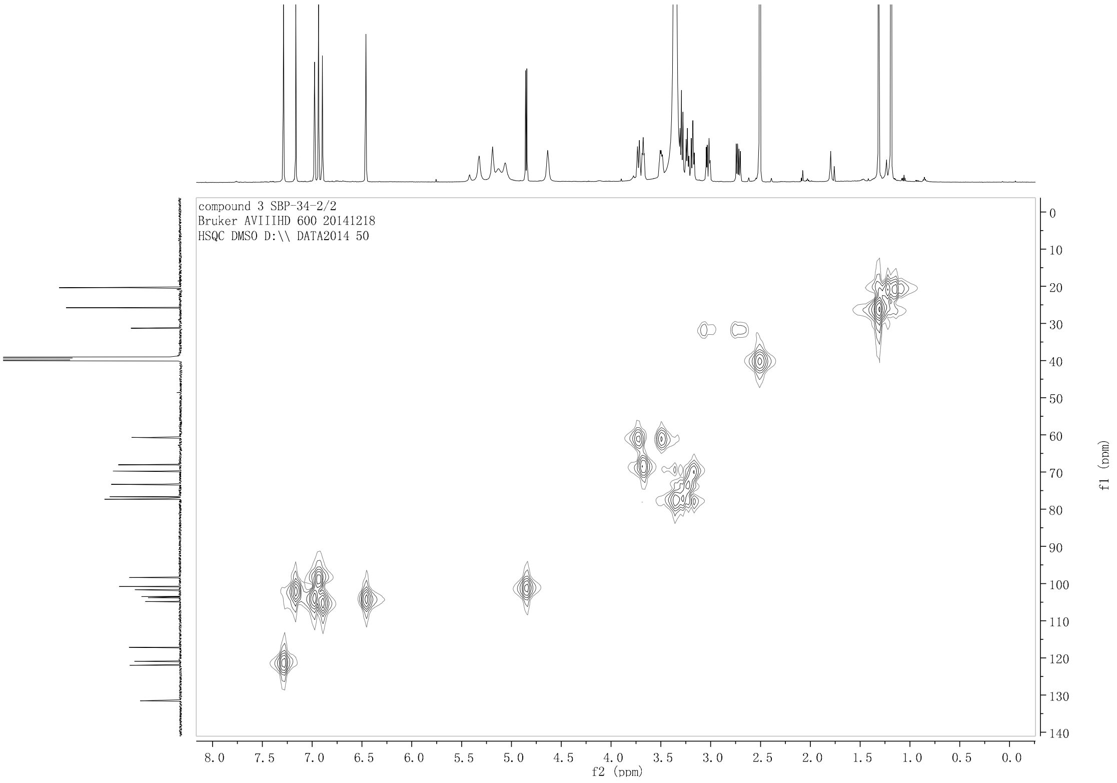

Figure SI-3c. HMQC spectra of compound **3**.

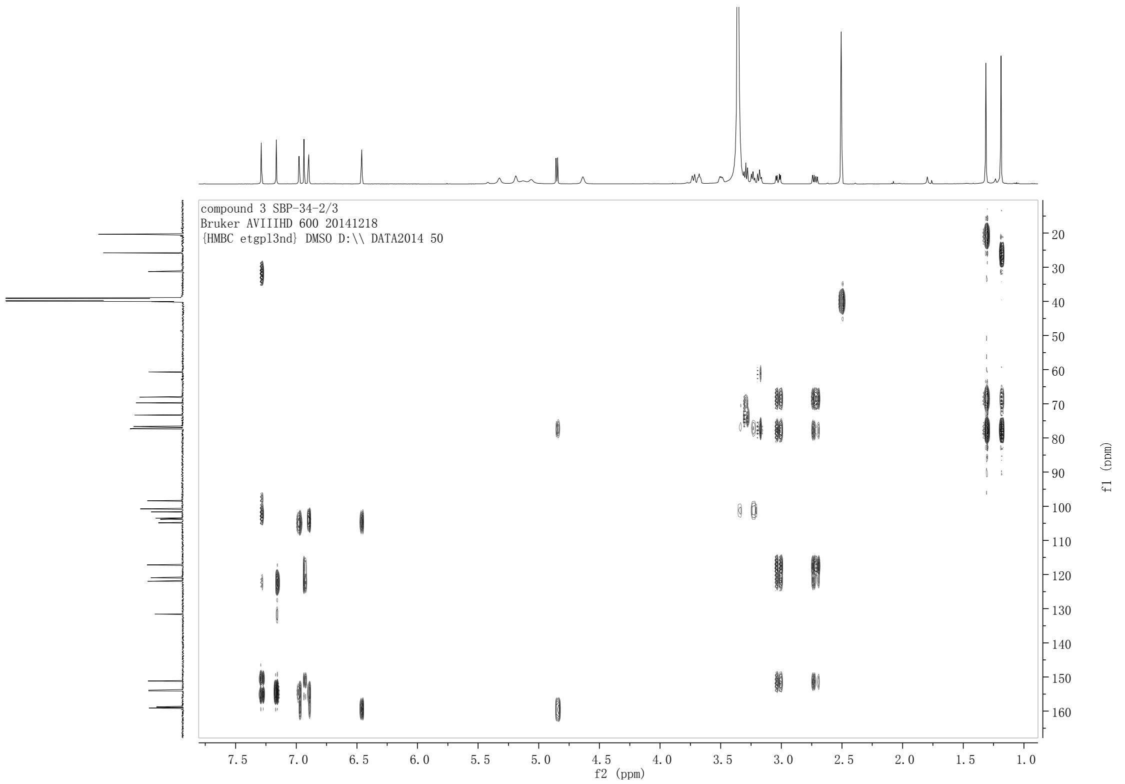

Figure SI-3d. HMBC spectra of compound **3**.

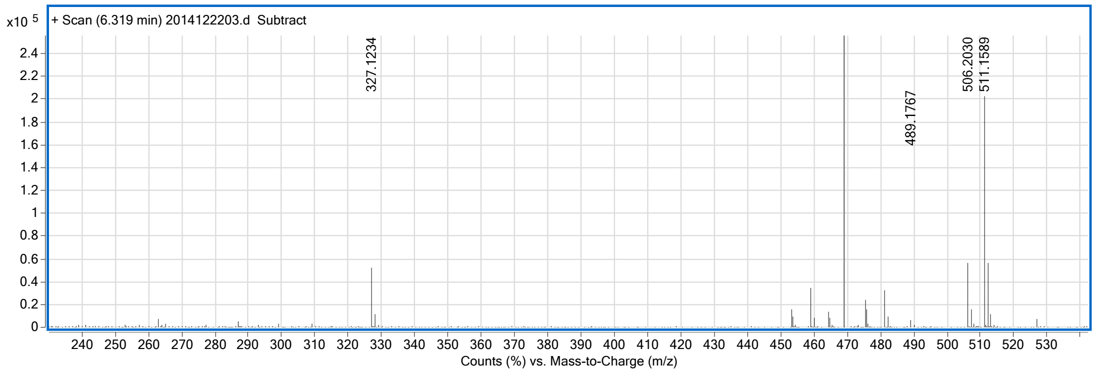

Figure SI-3e. (+)ESIMS spectra of compound **3**.

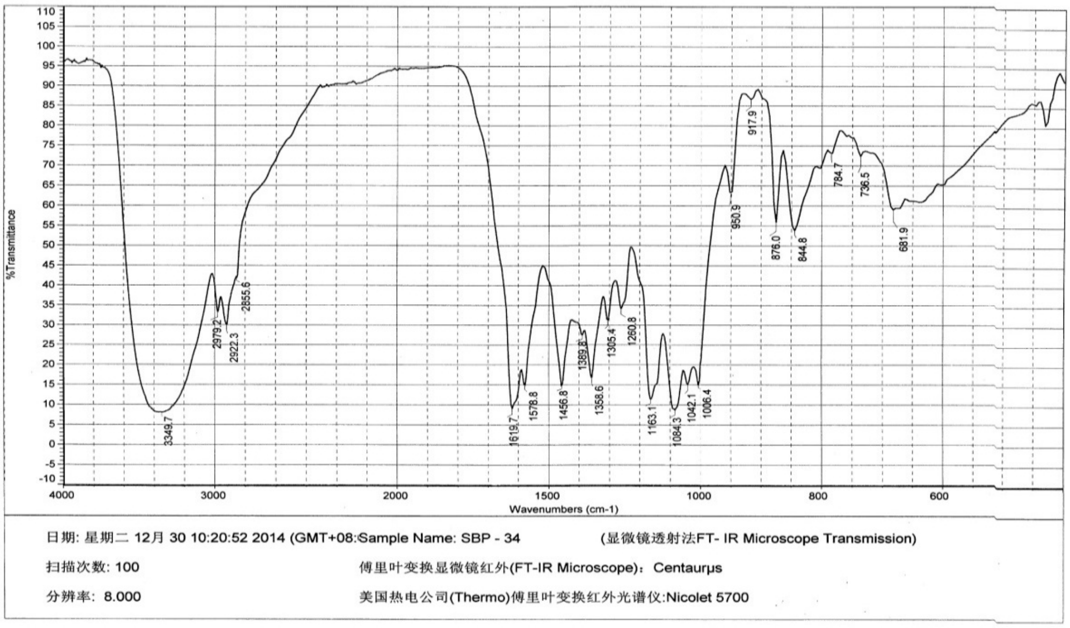

Figure SI-3f. IR spectra of compound **3**.

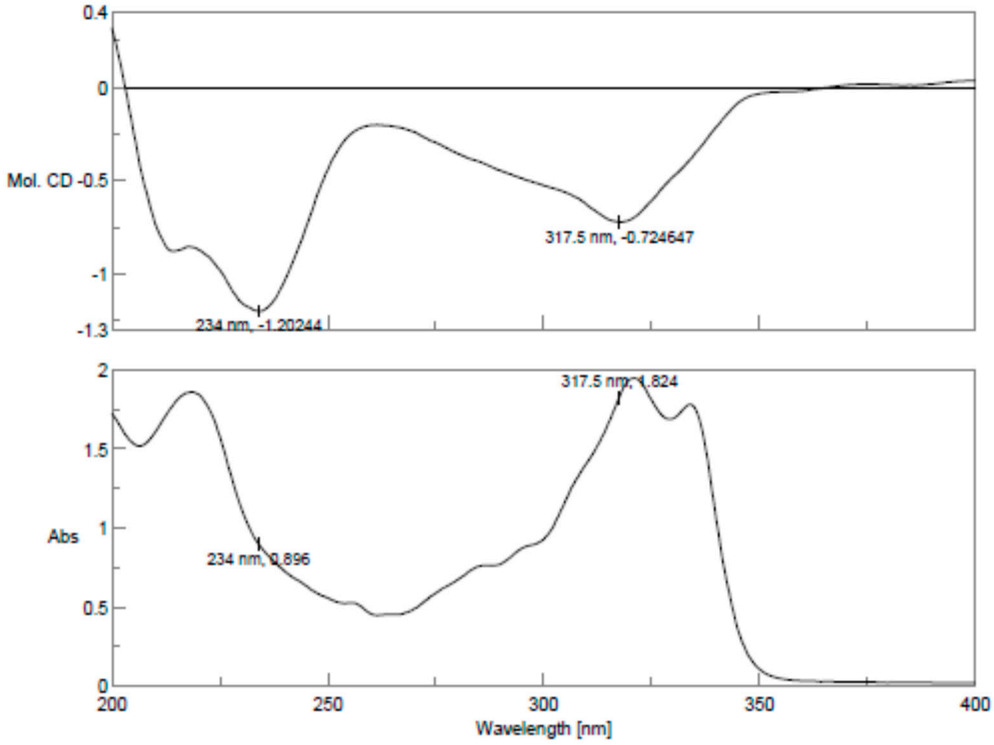

Figure SI-3g. CD and UV spectrum of compound **3**.

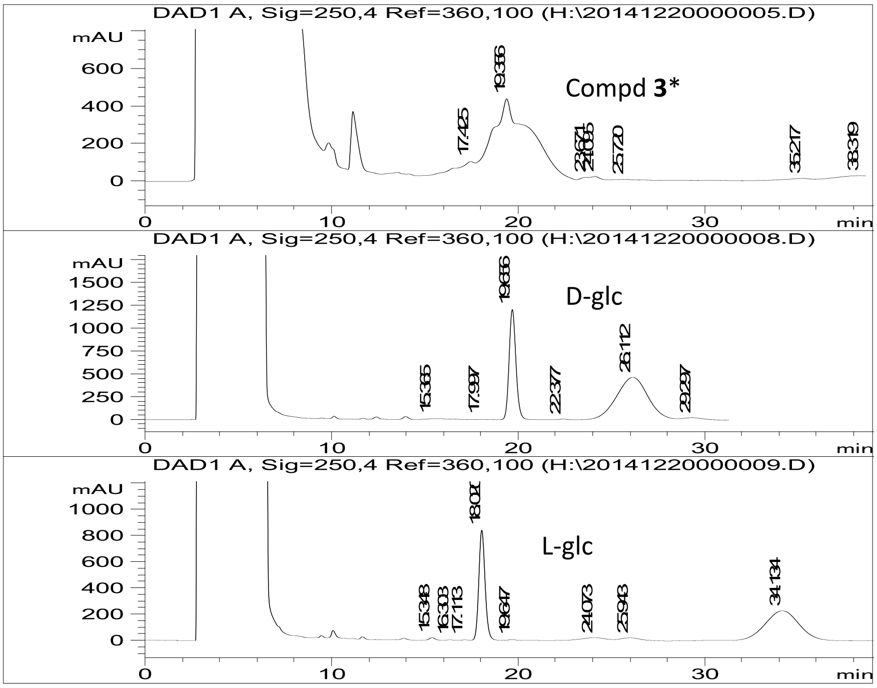

Figure SI-3h. HPLC of compound **3**, D-glucose and L-glucose.

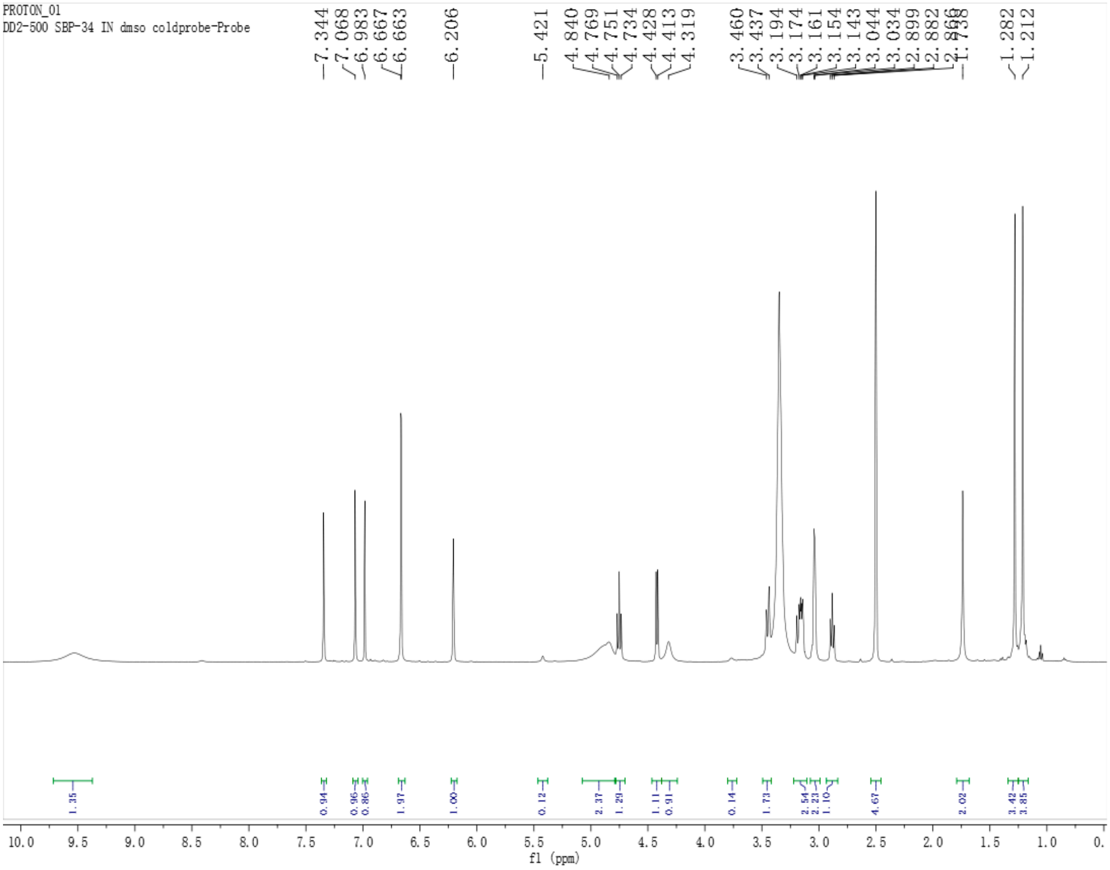

Figure SI-4a. ^{1}H NMR spectra of compound **4**.

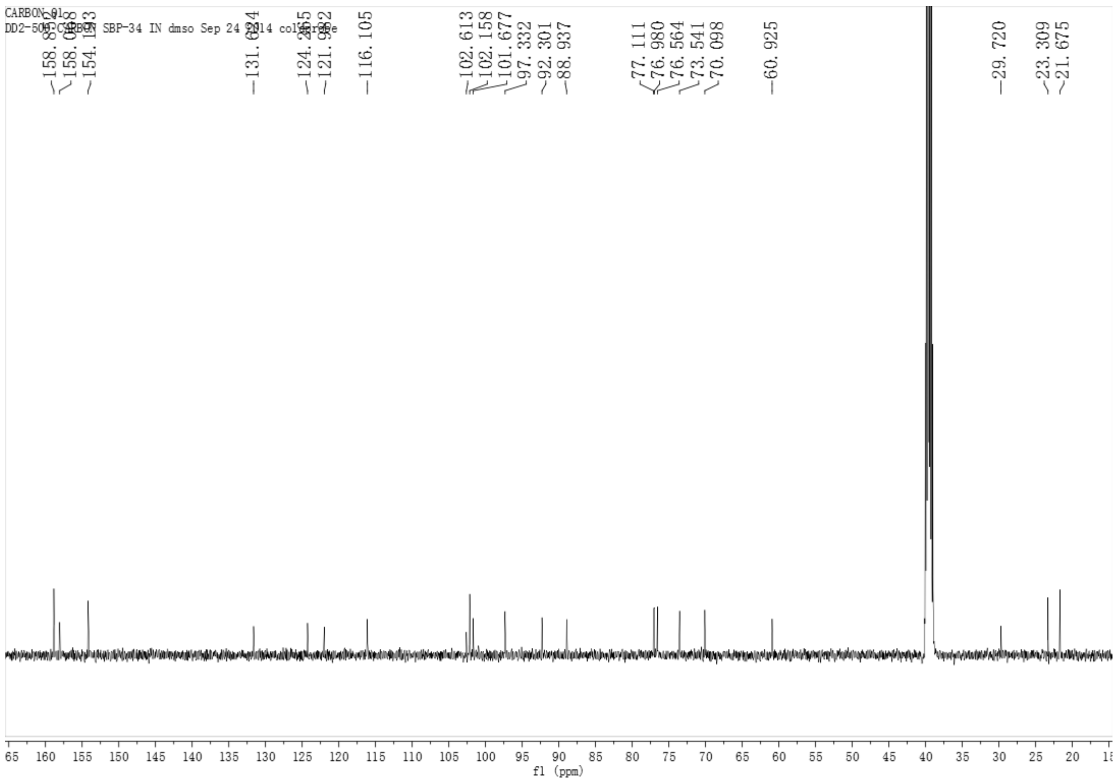

Figure SI-4b. ^{13}C NMR spectra of compound **4**.

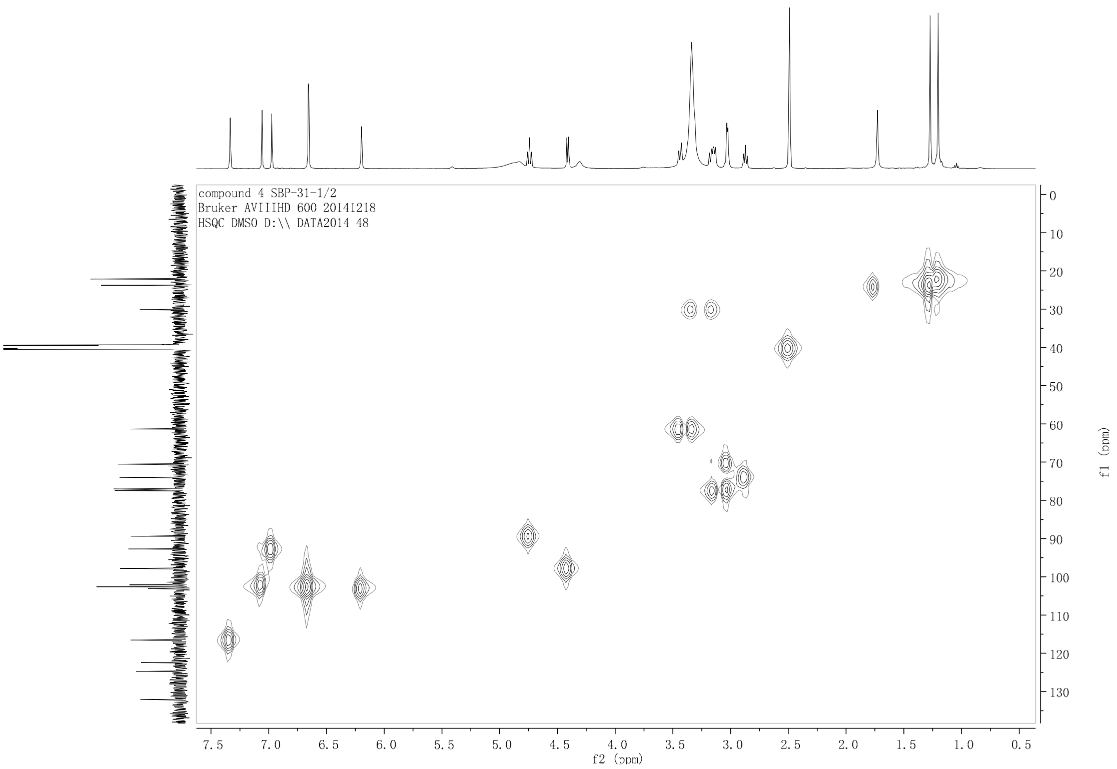

Figure SI-4c. HMQC spectra of compound **4**.

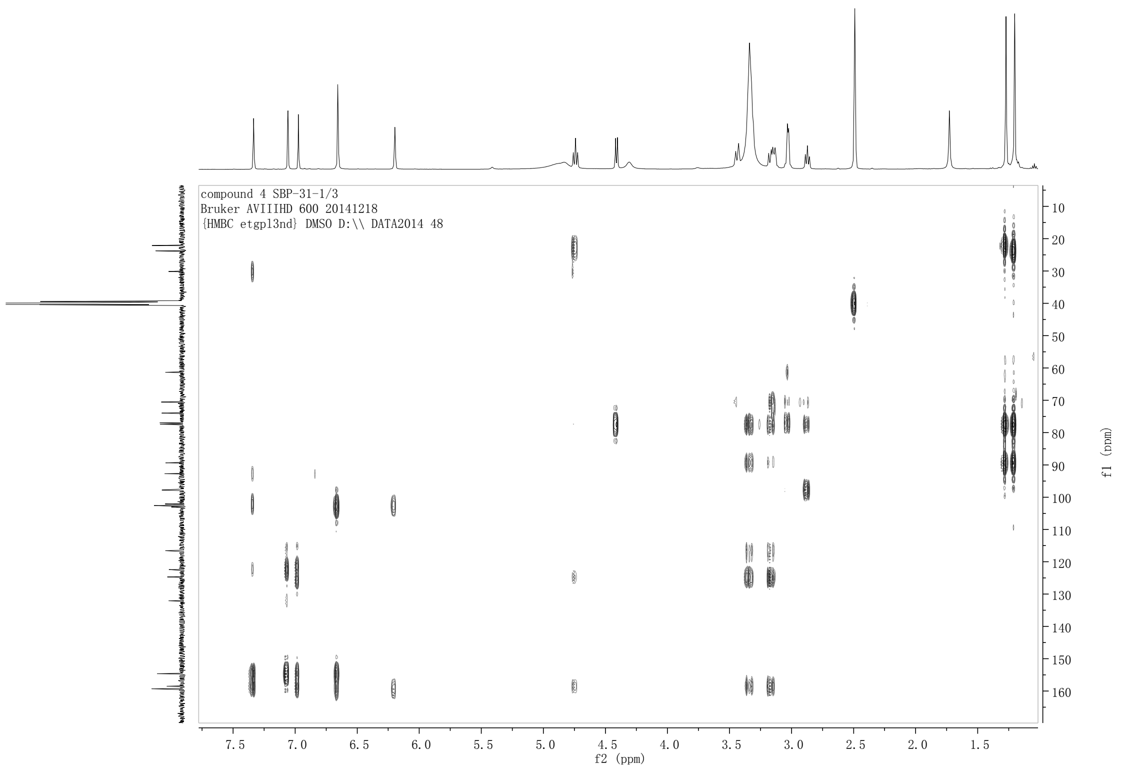

Figure SI-4d. HMBC spectra of compound **4**.

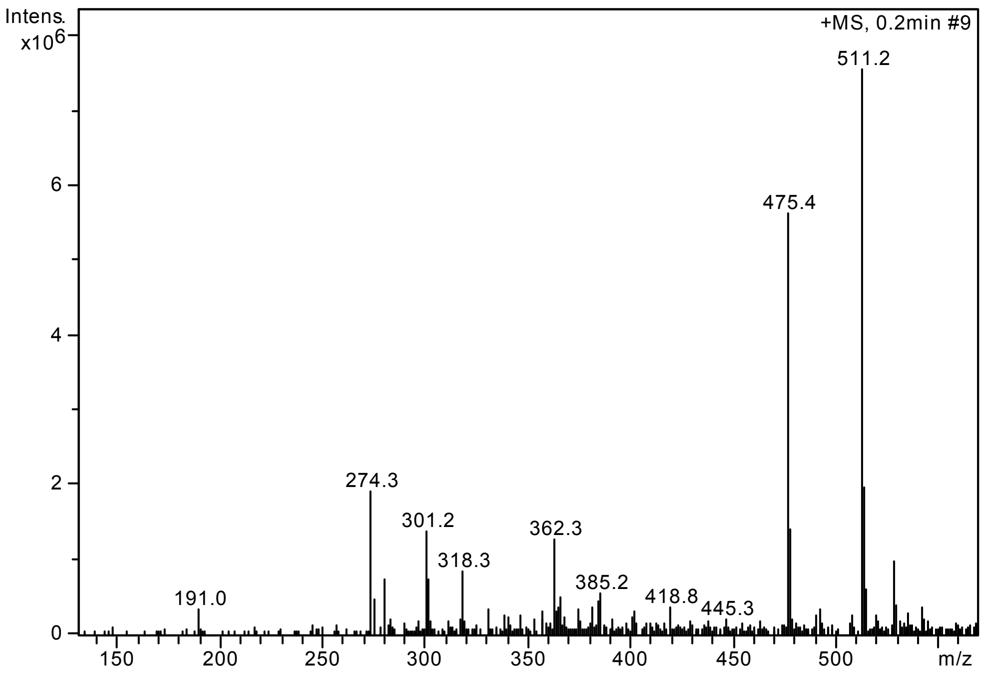

Figure SI-4e. (+)ESIMS spectra of compound **4**.

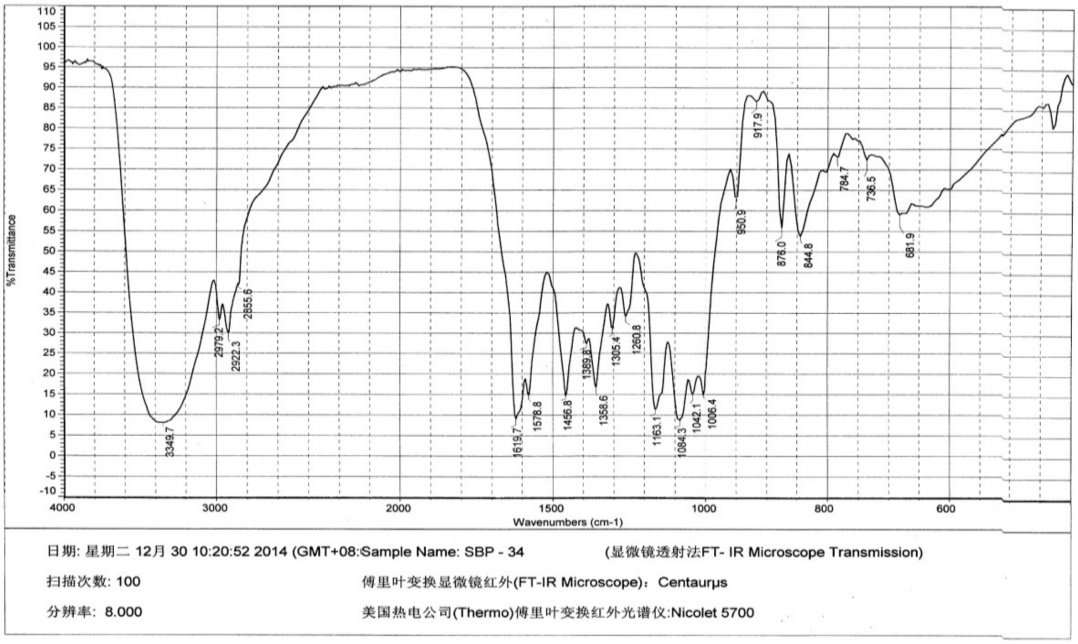

Figure SI-4f. IR spectra of compound **4**.

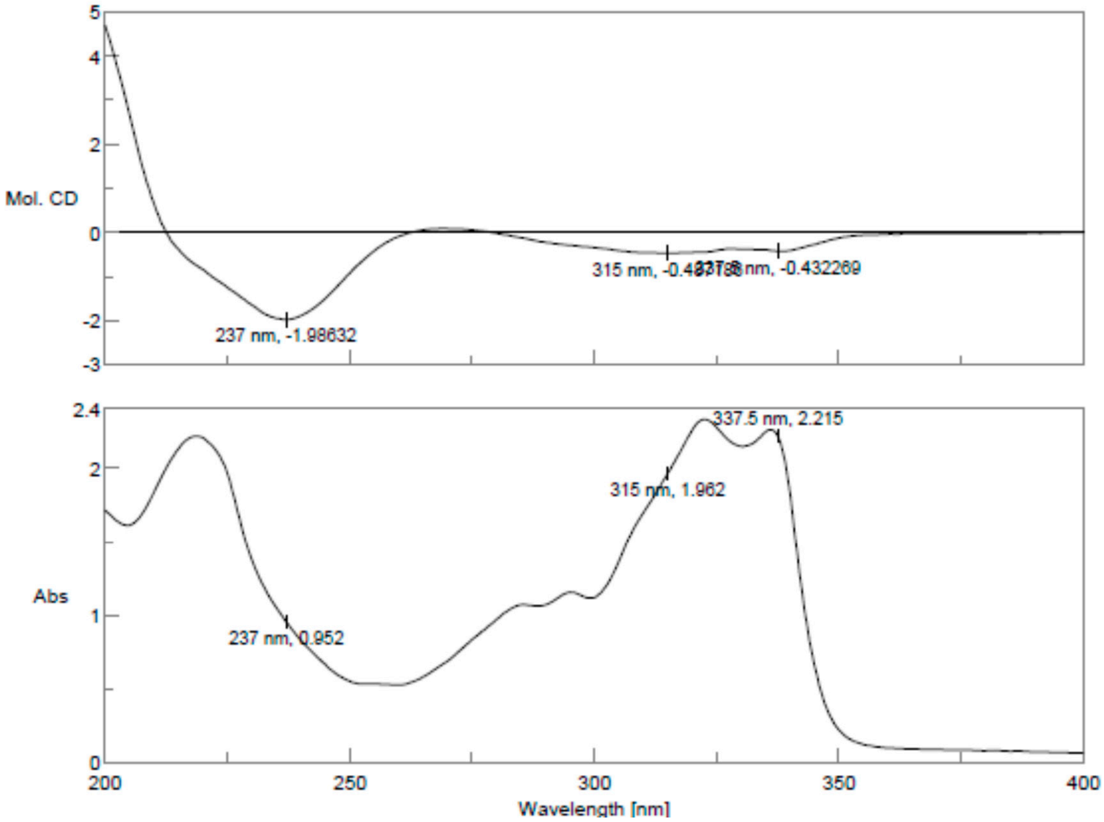

Figure SI-4g. CD and UV spectrum of compound **4**.

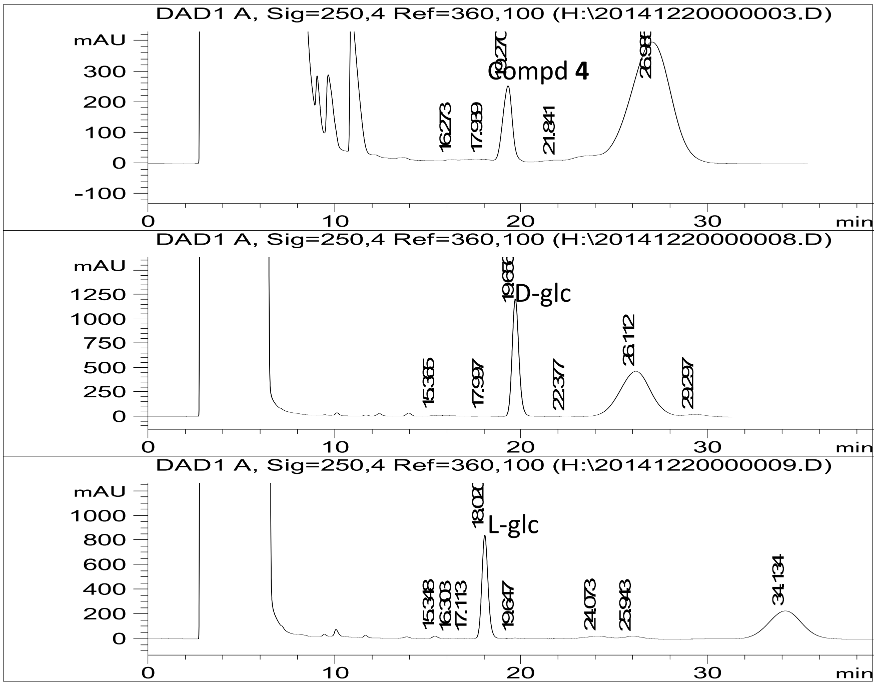

Figure SI-4h. HPLC of compound **4**, D-glucose and L-glucose.

Table SI-1. Optical rotation of aglycones **1a**–**4a**, moracin O (**5**), and P (**8**).

Compounds	$[\alpha]_D^{20}$ (*c* 0.1 MeOH)	$[\alpha]_D^{28}$ (*c* 0.04 MeOH)
1a	−0.0328 *	
2a	−0.0210 *	
3a	−0.0263 *	
4a	−0.0095 *	
Moracin O (**5**)	−5.2	−4.2
Moracin P (**8**)	−38.6	−15.9

* $[\alpha]_D^{20}$ can't be calculated due to the non-quantitative determination.

Calculated ECD of 1a and MO Analysis

Calculated ECD was carried out on *R* configuration of **1a**. Conformation search was done with the MMFF94 using the MOE software package (MOE2009.10, Chemical Computing Group, Montreal, QC, Canada). Calculated ECD was performed using TDDFT (Gaussian 09 B.01, Gaussian, Wallingford, CT, 2009) at B3LYP/6-31+G(d,p)//B3LYP/6-311+G(d,p) level for the configurations within an energy window of 5 kcal/mol. The conductor-like polarizable continuum model was used with MeOH (ε = 32.613) to take the solvent effects into consideration. The Boltzmann distribution was calculated based on the relative free energy (ΔG), and the final ECD (σ = 0.2 eV, UV shift = −15 nm) was simulated by using SpecDis (V1.64, University of Wuerzburg, Germany, 2015).

Table SI-2. The Boltzmann distribution of three conformations (C1–C3) of **1a**

Conformations of 1a	Δ*G* (Kcal/mol)	Boltzmann distribution (%)
C1	0.000	39.31
C2	0.349	21.80
C3	0.007	38.88

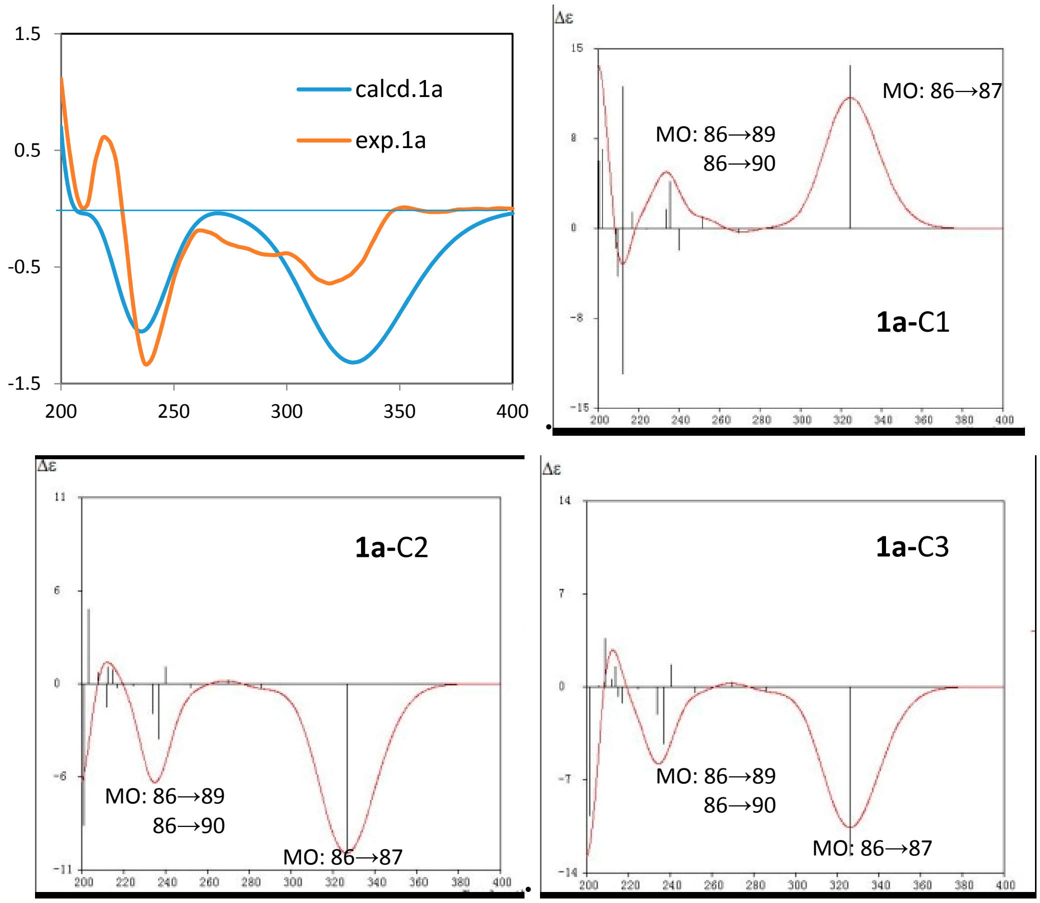

Figure SI-5a. Calculated ECD spectra of **1a**-C1–C3.

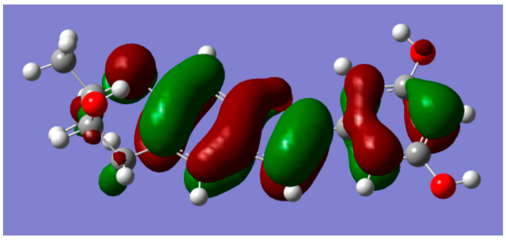
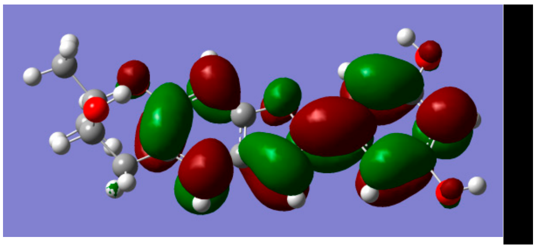

MO86: HOMO π of 2-benzylbenzofuran. MO87: LUMO π* of 2-benzylbenzofuran

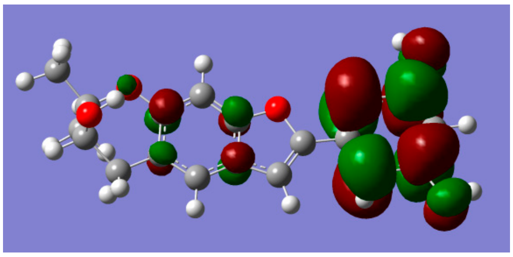
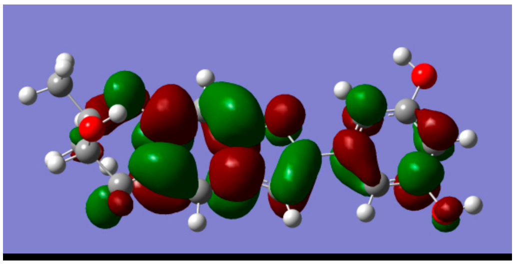

MO89: LUMO+2 π* of benzyl moiety. MO90: LUMO+3 π* of benzofuran moiety

Figure SI-5b. MO related with two Cotton effects of **1a**.

HPLC Profile of Cortex Mori Radicis and Their Main Components Identified by HPLC-MS

HPLC condition: The mobile solvent was: 0.1% formic acid (A) and B: MeOH (B). The gradient was: 10% B (0 min) → 100% B (30 min). The wavelength was 290 nm. The flow rate was 1.0 mL/min. The column was Previl C_{18} (Grace, 250 × 4.6mm, 5μ).

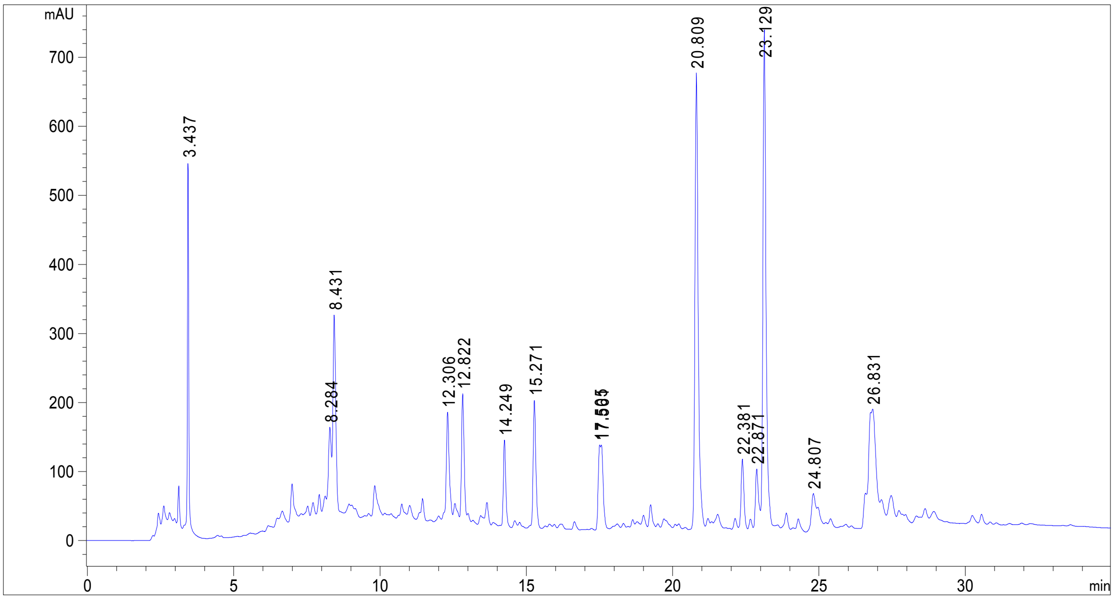

Figure SI-6. HPLC profile of Cortex Mori Radicis.

Table SI-3. Main components of Cortex Mori Radicis identified by HPLC-MS

R.T.	Area	Area %	M.W.	Name	Compound N.O.
3.44	1714.9	6.857	406		
8.28	832	3.327	568		
8.43	1941.7	7.763	----		
11.7	----	----	344	moracin R	7
12.31	1134.3	4.535	339		
12.82	1151.5	4.604	244	oxyresveratrol	6
13.1	----	----	489		2, 3, 4
13.8	----	----	458		1
14.25	724.8	2.898	458	mulberroside C	9
14.6	----	----	302	morin	14
15.27	1229.6	4.916	----		
17.50	629.5	2.517	326	moracin O	5
17.56	745.6	2.981	326	moracin P	8
18.1	----	----	420	morusin	15
18.3	----	----	562	isomulberrofuran G	11
18.6	----	----	562	mulberrofuran G	12
19.2	----	----	286	norartocarpetin	13
20.81	5046.5	20.177	708	Sanggenon D	17
22.38	673.5	2.693	690	cathayanins B	18
22.87	622.6	2.489	776		
23.13	5302.6	21.201	708	Sanggenon C	16
24.81	540.5	2.161	----		
26.83	2722.1	10.883	508		

The area % was calculated by the area of 290 nm.
